# PVC Nanoplastics Exposure Exacerbates Asthma through R‐Loop Accumulation and Subsequent STING Activation in Macrophages

**DOI:** 10.1002/advs.202502223

**Published:** 2025-09-08

**Authors:** Qimei Bao, Yixing Huang, Mingcong Deng, Chunkai Zhang, Dan Zu, Hanyi He, Yangchan Hu, Yuke Zhong, Chen Liang, Haidong Liu, Wumin Dai, Yanhua He, Yaoshu Teng, Ji Jing, Yin Shi, Xiangdong Cheng, Zu Ye

**Affiliations:** ^1^ Postgraduate training base Alliance of Wenzhou Medical University (Zhejiang Cancer Hospital) Hangzhou 310022 China; ^2^ Zhejiang University School of Medicine Hangzhou 310058 China; ^3^ Department of Otorhinolaryngology Hangzhou First People's Hospital Zhejiang University School of Medicine Hangzhou 310006 China; ^4^ School of Life Sciences Tianjin University Tianjin 300100 China; ^5^ Hangzhou Institute of Medicine (HIM) Chinese Academy of Sciences Hangzhou Zhejiang 310022 China; ^6^ Key Laboratory of Prevention Diagnosis and Therapy of Upper Gastrointestinal Cancer of Zhejiang Province Hangzhou 310022 China; ^7^ Zhejiang Provincial Research Center for Upper Gastrointestinal Tract Cancer Zhejiang Cancer Hospital Hangzhou 310022 China; ^8^ Department of Biochemistry and Department of Pulmonology Children's Hospital Zhejiang University School of Medicine Hangzhou 310058 China; ^9^ Guangxi Key Laboratory of Early Prevention and Treatment for Regional High Frequency Tumor Nanning 530021 China; ^10^ Key Laboratory of Early Prevention and Treatment for Regional High Frequency Tumor (Guangxi Medical University) Ministry of Education Nanning 530021 China

**Keywords:** asthma, PVC nanoplastics, R‐loops, RNASEH1, STING activation

## Abstract

Asthma is a chronic inflammatory respiratory disease influenced by genetic and environmental factors. Emerging evidence suggests that microplastics and nanoplastics (NPs) pose significant health risks. When inhaled, these tiny particles can accumulate in the lungs, triggering inflammation, oxidative stress, and other disruptions in pulmonary function. This study investigates the role of polyvinyl chloride (PVC) NPs, which are extensively used in products such as packaging, medical devices, and construction materials, in asthma pathogenesis. Using an ovalbumin (OVA)‐induced murine asthma model, it is demonstrated that PVC NPs exposure exacerbates airway hyperresponsiveness, increases inflammatory cell infiltration, and elevates inflammatory cytokine levels in the lungs. Further mechanistic studies reveal that PVC NPs suppress Ribonuclease H1 (RNASEH1), leading to RNA–DNA hybrid loop (R‐loop) accumulation and activation of the Cyclic GMP‐AMP Synthase (cGAS)‐Stimulator of Interferon Genes (STING) inflammatory pathway. The critical involvement of this pathway is confirmed using STING‐deficient mice, where pathway inhibition alleviates the inflammation exacerbated by PVC NPs exposure. These findings provide new insights into the potential role of NPs pollutants in modulating immune responses through R‐loop formation, linking PVC NPs to asthma pathogenesis. This study highlights the importance of addressing environmental exposure to NPs in asthma prevention and management and identifies potential molecular targets for therapeutic intervention.

## Graphical abstract

Polyvinyl chloride (PVC) nanoplastics (NPs) are shown to aggravate allergic airway inflammation in asthma by triggering R‐loop accumulation and activating the cGAS‐STING pathway in macrophages. This study reveals a novel immunotoxic mechanism linking environmental plastic exposure to asthma pathogenesis and highlights potential molecular targets for mitigating nanoplastic‐induced respiratory inflammation.

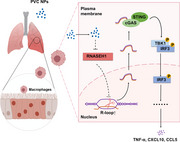

## 1 Introduction

Asthma is a complex, heterogeneous disease primarily characterized by airway inflammation, bronchial hyperresponsiveness, and reversible airflow obstruction, often accompanied by airway remodeling.^[^ ^1^ ^]^ Currently, asthma affects more than 300 million individuals globally, presenting a significant public health challenge due to its increasing prevalence and associated morbidity.^[^ ^2^ ^]^ The pathogenesis of asthma is multifactorial, involving an intricate interaction of genetic predisposition, environmental factors, and immune system activation.^[^ ^3^ ^]^ Environmental exposures, particularly to inhalants such as allergens, pollutants, and viral infections, have been identified as key contributors to the onset and exacerbation of asthma symptoms.^[^ ^3^ ^]^

In recent years, microplastic (MP) and nanoplastic (NP) pollution has emerged as one of the major environmental concerns.^[^ ^4^, ^6^, ^7^ ^]^ In particular, MPs and NPs have been shown to induce inflammatory responses, oxidative stress, and other physiological disruptions, posing significant health threats.^[^ ^8^, ^9^, ^10^, ^11^, ^12^ ^]^ Globally, ≈250 million tons of plastic products are produced annually, and a significant portion of these products degrade due to wear, weathering, and UV damage, resulting in the release of large quantities of plastic particles into the atmosphere.^[^ ^13^ ^]^ These particles are extremely small and lightweight, making them more likely to penetrate deep into the lungs. Studies have identified the accumulation of various plastic particles, including polypropylene (PP), polyethylene (PE), and polyvinyl chloride (PVC), in human lung tissue samples and bronchoalveolar lavage fluid (BALF), especially in patients with respiratory conditions.^[^ ^14^, ^15^, ^16^, ^17^ ^]^ Understanding the impact of microplastic exposure on human health, especially in relation to respiratory diseases such as asthma, is crucial for the development of effective protective strategies. Furthermore, compared to MPs, NPs demonstrate enhanced biological penetration capability owing to their smaller particle size and higher specific surface area, which not only facilitates greater bioaccumulation but may also lead to more severe health risks.^[^ ^18^, ^19^ ^]^ However, the molecular mechanisms underlying the inflammatory responses triggered by respiratory exposure to NPs remain poorly understood and require further investigation.

Among various plastics, PVC is one of the most widely produced plastics globally and constitutes a major component of microplastic pollution in indoor air.^[^ ^20^ ^]^ Compared with other polymers, PVC NPs have shown high bioactivity, inducing oxidative stress, DNA damage, and inflammatory cytokine release in human lung cells.^[^ ^7^, ^21^, ^22^, ^23^, ^24^ ^]^ Recent studies have indicated that exposure to NPs, including PVC‐derived particles, may contribute to the development and exacerbation of asthma. For instance, it has been shown that NPs can activate the NLRP3 inflammasome in macrophages, triggering the release of pro‐inflammatory cytokines such as IL‐1β and IL‐1α, which play key roles in amplifying the inflammatory response in asthma.^[^ ^11^ ^]^ Additionally, exposure to PVC dust has been linked to impaired lung function, further underscoring the potential risk posed by PVC particles to respiratory health.^[^ ^12^ ^]^ In vivo PVC exposure has been shown to aggravate airway inflammation in mouse lungs and increase systemic inflammation.^[^ ^16^, ^17^, ^24^ ^]^ Therefore, based on their environmental ubiquity, clinical relevance, and documented bioactivity, PVC NPs were selected in this study as representative airborne plastic particles to investigate their role in asthma‐like airway inflammation.

R‐loops are three‐stranded nucleic acid structures composed of an RNA‐DNA hybrid and a displaced single‐stranded DNA. Under normal conditions, the R‐loop plays essential physiological roles, including the regulation of gene transcription, replication, and genomic architecture.^[^ ^25^, ^26^, ^27^, ^28^, ^29^ ^]^ The enzyme ribonuclease H (RNase H) plays a crucial role in regulating R‐loop stability by degrading the RNA component of the hybrid, thereby preventing excessive accumulation and maintaining genomic integrity.^[^ ^30^, ^31^, ^32^, ^33^, ^34^ ^]^ Notably, persistent R‐loop accumulation can activate the cyclic GMP‐AMP synthase (cGAS)‐STING pathway, which senses cytoplasmic nucleic acids and initiates downstream inflammatory responses, including those involving type I interferons and NF‐kappa‐B (NF‐κB)‐dependent cytokines.^[^ ^25^, ^26^, ^30^, ^31^, ^32^, ^33^, ^34^, ^35^ ^]^ In macrophages, which are key effector cells in allergic airway inflammation, R‐loop‐driven STING activation has been shown to increase the secretion of inflammatory mediators and promote immune cell recruitment.^[^ ^30^, ^31^, ^32^, ^36^ ^]^ These immune effects mirror key features of asthma, including cytokine imbalance, airway remodeling, and immune cell infiltration. However, to date, no studies have directly investigated the role of R‐loop accumulation in the pathogenesis of asthma or allergic airway inflammation.

In this study, we sought to elucidate the immunological and molecular mechanisms by which PVC NPs aggravate allergic airway inflammation. Using a murine OVA‐induced asthma model, we found that intranasal PVC NPs exposure significantly worsened airway hyperresponsiveness and inflammatory cell infiltration. Notably, PVC NPs were found to accumulate in the lungs and localize within alveolar macrophages, which exhibited enhanced pro‐inflammatory cytokine production and chemokine‐mediated recruitment. Mechanistically, we demonstrate that PVC NPs induce abnormal accumulation of R‐loop structures in macrophages by downregulating RNASEH1, which in turn activates the cGAS‐STING signaling pathway. This activation is associated with increased production of pro‐inflammatory cytokines, including IL‐4, IL‐5, and IL‐13, alongside reduced IFN‐γ expression.^[^ ^37^, ^38^ ^]^ Importantly, both pharmacologic inhibition and genetic knockout of STING attenuated macrophage activation and cytokine release, supporting the central role of the R‐loop‐STING axis in mediating microplastic‐induced airway inflammation.

Together, our findings reveal a previously unrecognized immunological mechanism by which PVC NPs exacerbate asthma through R‐loop accumulation and STING‐mediated immune activation in lung macrophages. This work not only expands our understanding of how airborne microplastics impact respiratory health but also suggests potential molecular targets (e.g., STING and RNASEH1) for mitigating allergic airway disease.

## 2 Results

### 2.1 PVC NPs Characterization and Pro‐Inflammatory Effects in Allergic Asthma

**Figure 1:**
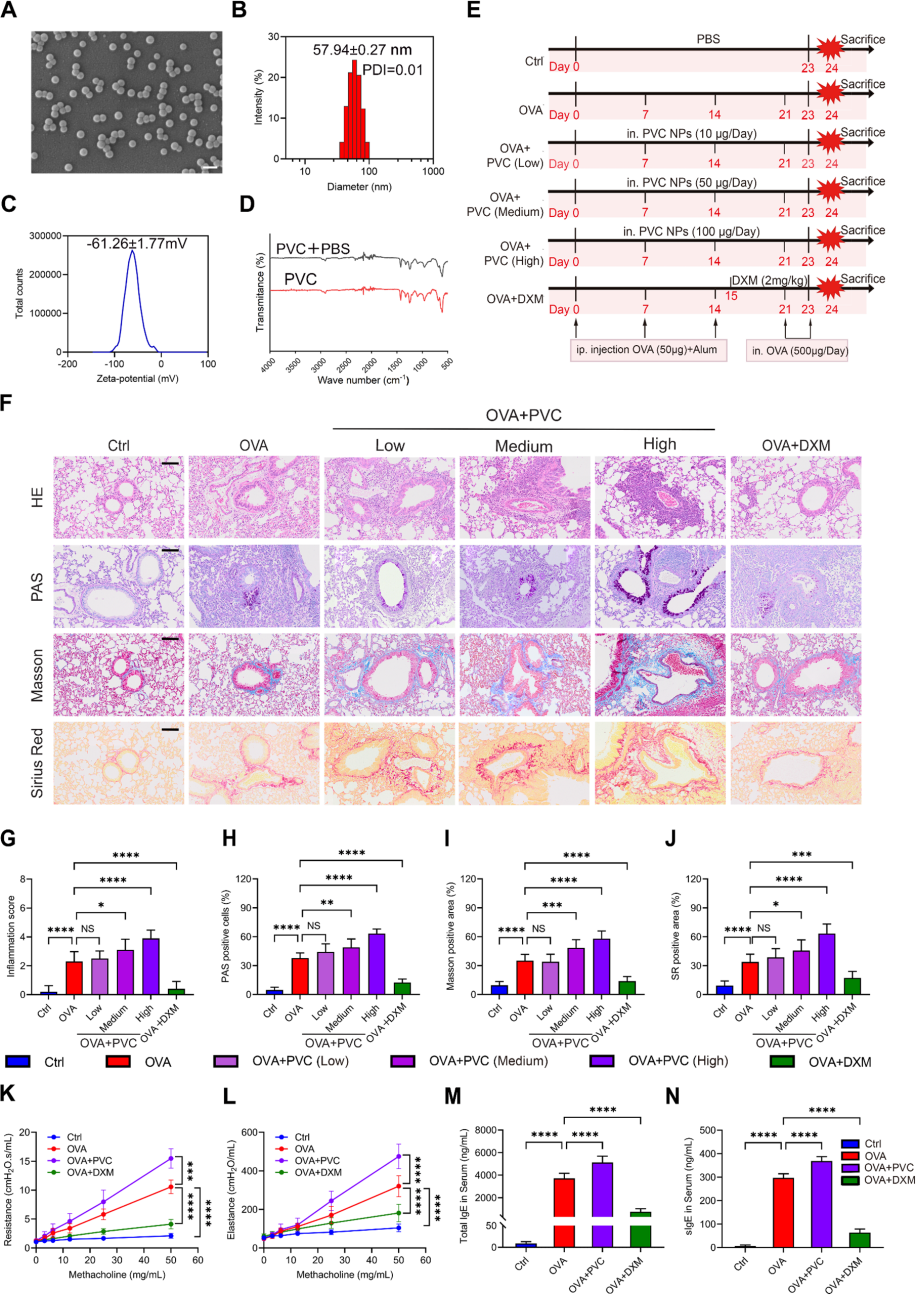
Characterization and pro‐inflammatory potential of PVC NPs in allergic asthma. A) Representative SEM images depicting the morphology of the PVC NPs (scale bar = 100 nm). B) Size distribution of the PVC NPs. C) Zeta potential of PVC NPs. D) FTIR absorbance spectra of fresh and PBS‐stored (72 h) PVC NPs. E) Establishment of the OVA‐induced allergic asthma model with PVC NPs exposure. F) Representative histopathological images of lung tissues from each group (scale bar = 200 µm), with corresponding quantitative scores for G) H&E staining, H) PAS staining, I) Masson's trichrome staining, and J) SR staining (*n* = 10). K,L) Changes in airway resistance (Rn) and airway elastance (Ers) in anesthetized mice in response to increasing methacholine concentrations (0–50 mg mL^−1^) 24 h after the final OVA challenge (*n* = 5). M,N) ELISA‐based quantification of total IgE and sIgE levels in mouse serum across experimental groups (*n* = 10). Statistical analysis was performed using one‐way (G–J,M,N) or two‐way (K,L) analysis of variance (ANOVA) followed by Tukey's multiple comparison test. The data are presented as the mean ± SD; *p < 0.05, **p < 0.01, ***p < 0.001, ****p < 0.0001, NS, not significant.

The morphological characteristics of the synthesized PVC NPs were analyzed via transmission electron microscopy (TEM) and scanning electron microscopy (SEM). Electron microscopy observations revealed that the PVC NPs exhibited a uniform spherical morphology with a geometric diameter of ≈50 nm (**Figure** **1**A; Figure S1, Supporting Information). Dynamic light scattering analysis demonstrated that the nanoparticles had a hydrodynamic diameter of 57.94 ± 0.27 nm (polydispersity index (PDI) = 0.01) (Figure 1B) and a zeta potential of −61.26 ± 1.77 mV in PBS (Figure 1C). These results collectively indicate that the prepared PVC NPs possess excellent monodispersity and colloidal stability. To evaluate the chemical stability of the nanoparticles, we performed Fourier transform infrared spectroscopy (FTIR) to compare freshly prepared PVC NPs with those incubated in PBS at 37 °C for 72 h (Figure 1D). The FTIR spectra displayed characteristic PVC NPs absorption peaks but no detectable signals corresponding to common plasticizers (e.g., phthalates) or stabilizers. Notably, no significant differences were observed between the FTIR spectra before and after incubation, confirming that the PVC NPs maintain their chemical structural integrity in the buffer system.

As described previously,^[^ ^39^ ^]^ an allergic asthma model was established through OVA sensitization and subsequent challenge. To assess the potential impact of nasal PVC NPs exposure on the progression of OVA‐induced allergic asthma, the mice in the asthma group were subjected to daily nasal instillation of either the presence or absence of PVC NPs for 24 consecutive days (Figure 1E). We next systematically evaluated the effects of PVC NPs exposure on pulmonary pathological alterations in an OVA‐induced murine asthma model. Hematoxylin and eosin (H&E) staining results showed that compared with the control mice, OVA‐challenged mice exhibited typical inflammatory damage in airways, including significant inflammatory cell infiltration, epithelial cell shedding, airway wall thickening, and lumen narrowing (Figure 1F,G). Notably, these injuries were obviously aggravated in a dose‐dependent manner in the PVC NPs‐exposed groups. Periodic Acid‐Schiff (PAS) staining analysis further demonstrated that both goblet cell hyperplasia and mucus secretion increased significantly with increasing doses of PVC NPs. Masson's trichrome staining and Sirius Red (SR) staining also confirmed that PVC NPs exposure enhanced peribronchial collagen deposition (Figure 1H‐J). Under polarized light microscopy, type I collagen fibers appear red or orange‐yellow, whereas type III collagen fibers appear green.^[^ ^40^ ^]^ Excessive subepithelial deposition of type I and III collagen correlates with asthma severity and irreversible structural remodeling.^[^ ^41^ ^]^ The OVA+PVC group showed increased proportions of type I collagen fibers (Figure S1B,C, Supporting Information), which may be associated with airway remodeling and loss of tissue elasticity. These histopathological findings demonstrate that PVC NPs significantly exacerbate OVA‐induced asthma progression by aggravating airway inflammation, promoting mucus hypersecretion, and inducing collagen deposition.

Through systematic histopathological evaluation, we observed that PVC NPs exposure dose‐dependently exacerbated pulmonary and airway pathological injuries in asthmatic mice. Based on this established dose‐response relationship, we selected the high‐dose PVC NPs exposure group demonstrating the most representative pathological alterations as the experimental intervention condition in subsequent investigations to comprehensively elucidate the potential toxic effects of PVC NPs on asthma pathogenesis. Asthma is characterized by airway hyperresponsiveness.^[^ ^1^ ^]^ In this study, we sought to determine whether PVC NPs exposure would exacerbate airway hyperresponsiveness in asthmatic mice. In the absence of PVC NPs exposure, the asthma group demonstrated elevated airway resistance and elastance compared with the control group, and DXM treatment significantly reduced airway hyperresponsiveness, confirming proper model establishment and accurate measurements. However, following PVC NPs exposure, the results indicated a marked exacerbation of airway hyperresponsiveness in asthmatic mice (Figure 1K,L). Quantitative ELISA analysis of murine serum samples demonstrated that, compared with the asthma model group, the PVC NPs‐exposed intervention group exhibited significantly elevated immunoglobulin levels, characterized by marked increases in both total IgE and allergen‐specific IgE concentrations (Figure 1M,N).

To further characterize the systemic effects of allergic airway inflammation, we assessed behavioral manifestations in asthmatic mice. We tracked the movement trajectories of the mice for a duration of 10 min (Figure S1D, Supporting Information) and quantified the total distance traveled (Figure S1E, Supporting Information). The mice in the control group (Ctrl) traveled the longest distance, followed by those in the OVA+DXM group. In contrast, the mice in the OVA group traveled less, and those in the OVA+PVC group traveled the least, indicating a further reduction in physical activity associated with PVC NPs exposure. Additionally, compared with the OVA group, the OVA+PVC group exhibited significantly increased stationary time (Figure S1F, Supporting Information). Although there was no significant difference in the frequency of upright postures between the OVA and OVA+PVC groups, a downward trend in the frequency of upright postures was observed in the OVA+PVC group (Figure S1G, Supporting Information). We also quantified nose‐wiping behavior, a well‐established surrogate for airway irritation in allergic airway inflammation.^[^ ^38^, ^42^ ^]^ Notably, during the same time period, mice in the OVA group exhibited a higher frequency of nose‐wiping behavior compared to the Ctrl group, with DXM treatment effectively mitigating this symptom. However, PVC NPs exposure further aggravated the incidence of nose‐wiping behavior, indicating that PVC NPs exposure exacerbates allergic responses and increases discomfort in asthmatic mice (Figure 1H). These findings collectively suggest that PVC NPs exposure reduces physical activity and further exacerbates the behavioral manifestations associated with allergic asthma in mice.

### 2.2 PVC NPs Exposure Increases the Levels of Immune Cells and Pro‐Inflammatory Factors in BALF

**Figure 2:**
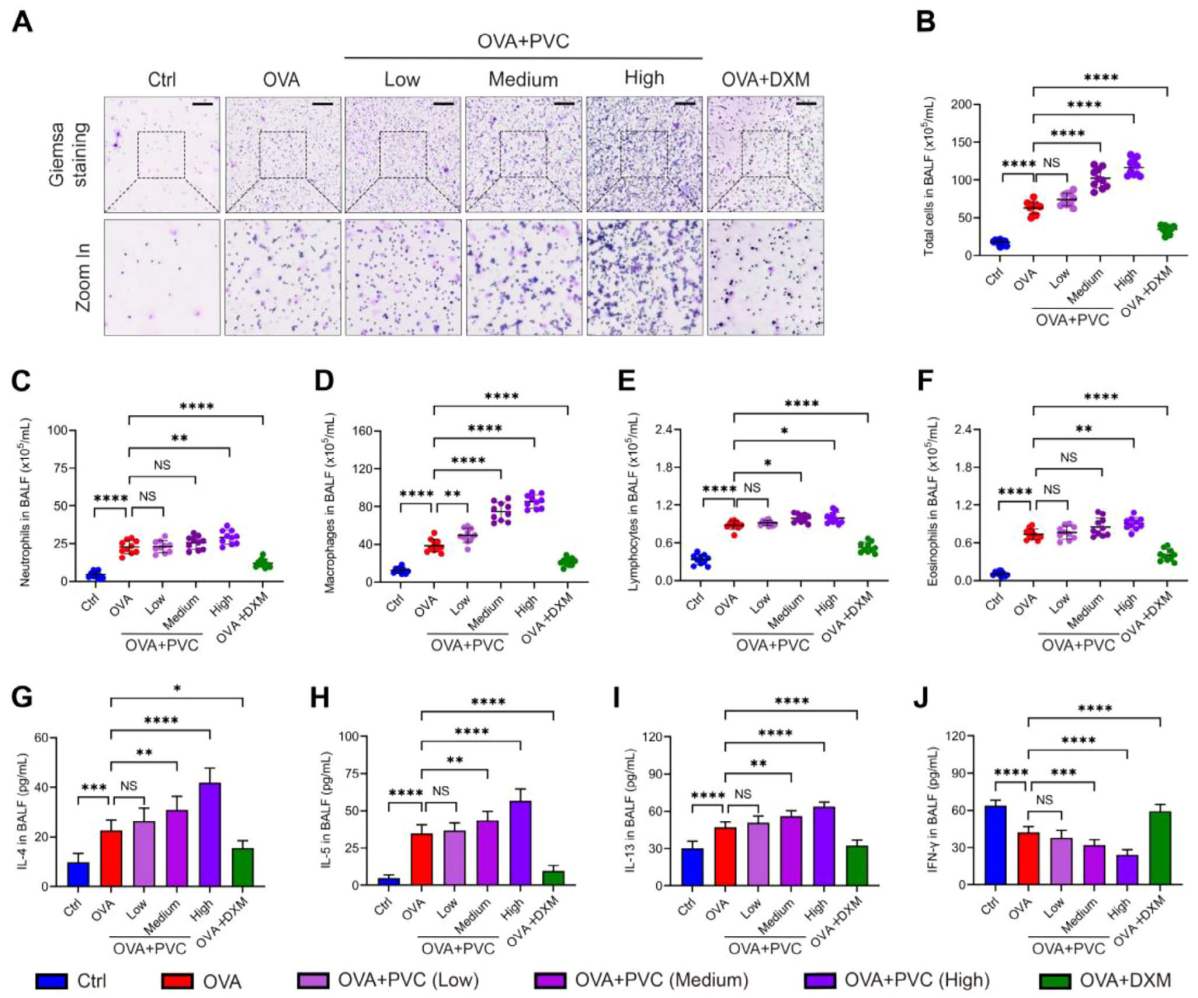
PVC NPs increase the levels of inflammatory cells and pro‐inflammatory factors in the BALF. A) Representative images of BALF smeared with Wright–Giemsa stain from each group of mice (scale bar = 200 µm). B–F) Quantitative analysis of total inflammatory cells (B), neutrophils (C), macrophages (D), lymphocytes (E), and eosinophils (F) in the BALF of each group of mice (*n* = 10). G–J) Quantitative analysis of the IL‐4 (G), IL‐5 (H), IL‐13 (I), and IFN‐γ (J) levels in the BALF of each group of mice via ELISA (*n *= 10). Statistical analysis was performed using one‐way ANOVA followed by Tukey's multiple comparison test. The data are presented as the mean ± SD; *p < 0.05, **p < 0.01, ***p < 0.001, ****p < 0.0001, NS, not significant.

Following the observation of airway structural damage and remodeling, we further examined the impact of PVC NPs exposure on the inflammatory response and immune profile in the OVA‐induced asthma model. After preparing cell smears from the BALF of the mice, Wright–Giemsa staining was performed (**Figure** **2**A), and microscopic observation and counting revealed a significant increase in the total cell count in the BALF of OVA‐induced asthmatic mice, with further increased after PVC NPs exposure (Figure 2B). The numbers of neutrophils, macrophages, lymphocytes, and eosinophils were counted and found to be increased (Figure 2C–F), with macrophages showing the most pronounced increase. Subsequent flow cytometric immunophenotyping of murine BALF cells revealed a marked increase in multiple immune cell subsets following PVC NPs exposure, including macrophages (CD11b+F4/80+), neutrophils (CD11b+Ly6G+), dendritic cells (CD11c+MHCII+), monocytes (CD11b+Ly6C+), and various T lymphocyte populations (CD3+, CD4+, CD8+) (Figure S2A–C, Supporting Information). Similarly, PVC NPs exposure induced particularly pronounced accumulation of macrophages. These findings demonstrate that PVC NPs exposure induces widespread recruitment and activation of both innate and adaptive immune cells in the lungs, thereby significantly amplifying the inflammatory response. Notably, macrophage recruitment and activation are particularly prominent, as these cells serve as key mediators of airway inflammation in asthma.

ELISA quantification of cytokines in murine BALF revealed significant perturbations in pulmonary immune homeostasis. PVC NPs exposure induced a marked increase in Th2 cytokines (IL‐4, IL‐5, and IL‐13) with concomitant suppression of the Th1 cytokine IFN‐γ (Figure 2G–J). Flow cytometric analysis further demonstrated significant expansion of CD4⁺ T cell populations. These findings collectively indicate that PVC NPs exposure activates a characteristic immunopathological signature of asthma.

### 2.3 PVC NPs Induce Cytotoxicity and Promote Inflammation in Macrophages

**Figure 3:**
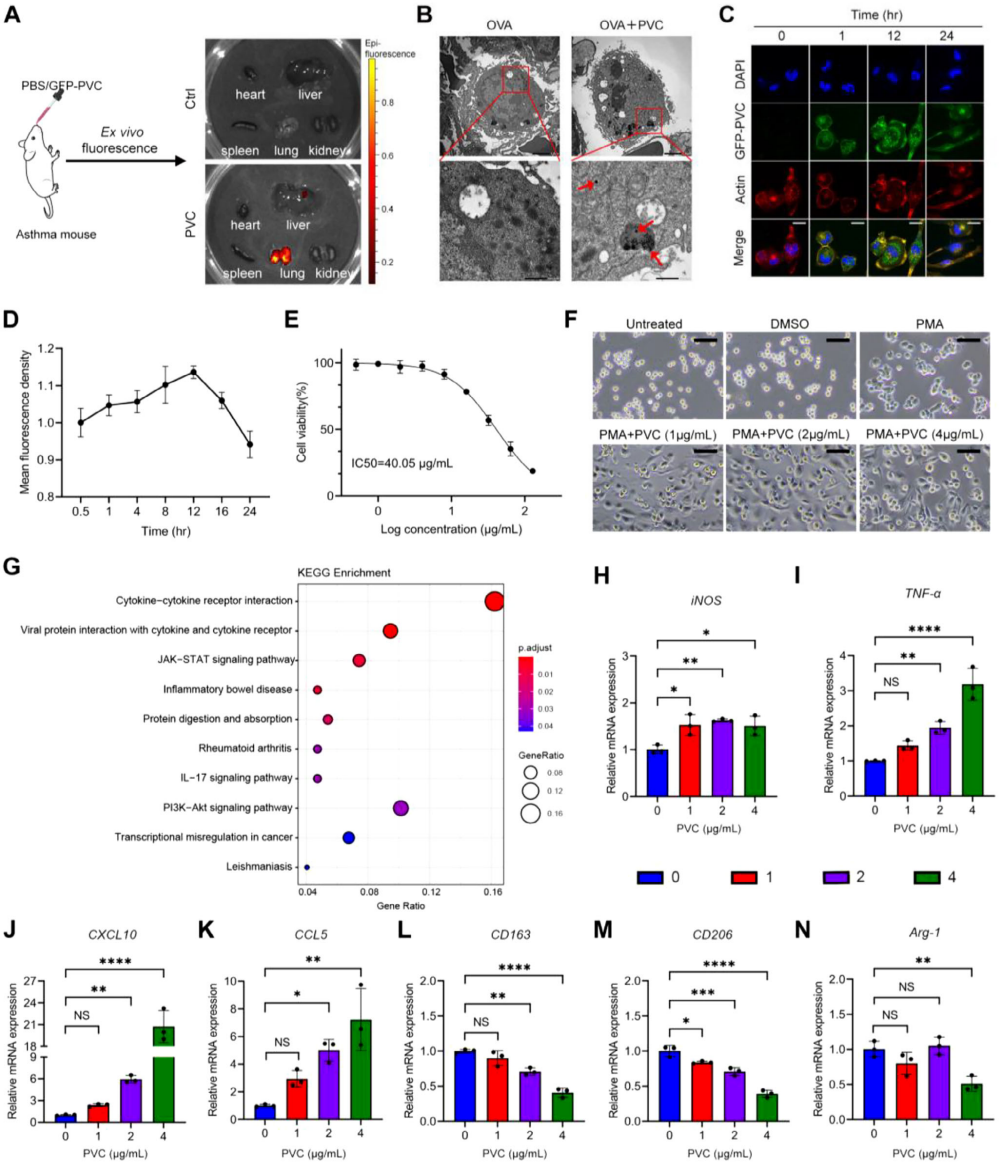
PVC NPs trigger macrophage cytotoxicity and pro‐inflammatory activation. A) Tissue distribution of PVC NPs in the hearts, livers, spleens, lungs, and kidneys following inhalation exposure. B) TEM images of the internalized PVC NPs in pulmonary macrophages (scale bar = 2 µm for upper panels; scale bar = 500 nm for lower panels). The PVC NPs particles are indicated by red arrows. C) Representative composite fluorescence images showing the entry of PVC‐GFP NPs into THP‐1‐derived macrophages. The PVC NPs, actin, and nuclei were labeled with GFP, actin‐tracker (red), and DAPI (blue), respectively (scale bar = 20 µm). D) Quantitative analysis of the relative fluorescence intensity of PVC‐GFP NPs at various time points across multiple fields of view (*n* = 4). E) The toxicity of different concentrations of PVC NPs on THP‐1 cells was assessed via a CCK8 assay. IC50 = 40.05 µg mL^−1^. F) After THP‐1 cells were induced to adhere and differentiate into macrophages with PMA, representative images of macrophage morphological changes were observed under a light microscope after treatment with different concentrations of PVC NPs for 48 h (scale bar = 20 µm). G) KEGG analysis for RNA‐seq of PVC NPs ‐treated or untreated THP‐1‐derived macrophages. H–N) Levels of iNOS mRNA (H), TNF‐α mRNA (I), CXCL10 mRNA (J), CCL5 mRNA (K), CD163 mRNA (L), CD206 mRNA (M), and Arg‐1 mRNA (N) in THP‐1‐derived macrophages treated with different concentrations of PVC NPs for 48 h, as measured by RT‐qPCR (*n* = 3). Statistical analysis was performed using one‐way ANOVA followed by Tukey's multiple comparison test. The data are presented as the mean ± SD; *p < 0.05, **p < 0.01, ***p < 0.001, ****p < 0.0001, NS, not significant.

To delineate the in vivo distribution characteristics of PVC NPs in asthmatic models, we administered fluorescently labeled PVC NPs to sensitized asthmatic mice via intranasal instillation. *Ex vivo* organ imaging analysis demonstrated pronounced pulmonary‐specific accumulation of PVC NPs (**Figure** **3**A). TEM ultrastructural examination further confirmed the internalization of characteristic 50 nm PVC NPs within pulmonary macrophages (Figure 3B, indicated by red arrows).

Given that PVC NPs exposure notably alters the pulmonary immune environment, we focused on macrophages because of the significant changes in their infiltration and activation observed in response to PVC NPs. Macrophages play a central role in immune responses and inflammation, making them key targets for understanding the molecular and cellular effects of PVC NPs. To assess the cytotoxicity of PVC NPs in macrophages, we first performed a CCK8 assay using THP‐1‐derived macrophages. The PVC NPs demonstrated significant cytotoxicity, with an IC50 value of 40.05 µg mL^−1^ (Figure 3E). Therefore, we used lower concentrations of PVC NPs in subsequent experiments to minimize cell toxicity. Next, we investigated whether PVC NPs could enter macrophages. We treated THP‐1‐derived macrophages with GFP‐labeled PVC NPs (50 nm) and found that PVC NPs were internalized in large quantities within 1 hour, with peak internalization occurring at 12 h, followed by a gradual decrease in the number of intracellular NPs by 24 h (Figure 3C,D). These findings suggest that the PVC NPs are efficiently internalized by macrophages. Confocal imaging confirmed that the PVC NPs accumulated significantly within the macrophages, highlighting their cellular uptake. To investigate the functional impact of PVC NPs on macrophages, we co‐cultured PVC NPs with PMA‐induced THP‐1 macrophages for 48 h. Macrophage morphology was significantly altered in a dose‐dependent manner, indicating that PVC NPs exposure may activate these cells (Figure 3F). Meanwhile, at a concentration of 4 µg mL^−1^ PVC NPs, macrophage morphology also exhibited dynamic changes over time, with distinct morphological alterations observable after 48 h of culture (Figure S3A, Supporting Information). These morphological changes suggest that PVC NPs have the potential to activate macrophages, which could contribute to the inflammatory response.

Further investigations into the molecular mechanisms revealed by PVC NPs exposure were conducted through RNA sequencing (RNA‐seq), which compared the gene expression profiles of PVC NPs‐treated and untreated THP‐1 macrophages. KEGG enrichment analysis revealed that several immune‐related pathways, including cytokine–cytokine receptor interactions and the JAK‐STAT signaling pathway, were significantly impacted (Figure 3G). Heatmap analysis of selected significantly altered gene expression patterns was performed, with subsequent validation via quantitative real‐time polymerase chain reaction (RT‐qPCR) (Figure S4A–M, Supporting Information). These findings suggest that PVC NPs exposure may affect key immune pathways involved in macrophage activation. Consistently, we observed dose‐dependent changes in the mRNA expression of several markers and cytokines in THP‐1‐derived macrophages. The mRNA levels of pro‐inflammatory markers, such as iNOS, TNF‐α, CXCL10, and CCL5, were significantly upregulated after PVC NPs exposure, while the expression of anti‐inflammatory markers, such as CD163, CD206, and Arg‐1, was decreased (Figure 3H–N). These changes indicate that PVC NPs exposure may promote a pro‐inflammatory phenotype in macrophages, shifting the balance toward a more inflammatory response. Transwell migration assays revealed that the chemotaxis of THP‐1‐derived macrophages was significantly higher in BALF from asthmatic mice than in that from control mice, with PVC NPs exposure further augmenting this chemotactic activity, suggesting that PVC NPs may promote macrophage recruitment by increasing chemokine secretion in BALF (Figure S5A,B, Supporting Information).

In conclusion, our results suggest that PVC NPs induce cytotoxicity and activate macrophages, leading to alterations in key immune pathways. These findings support the notion that PVC NPs exposure may drive macrophage activation and contribute to the exacerbation of inflammation in asthma, potentially influencing disease progression and severity.

### 2.4 PVC NPs Activate the cGAS‐STING Pathway

**Figure 4:**
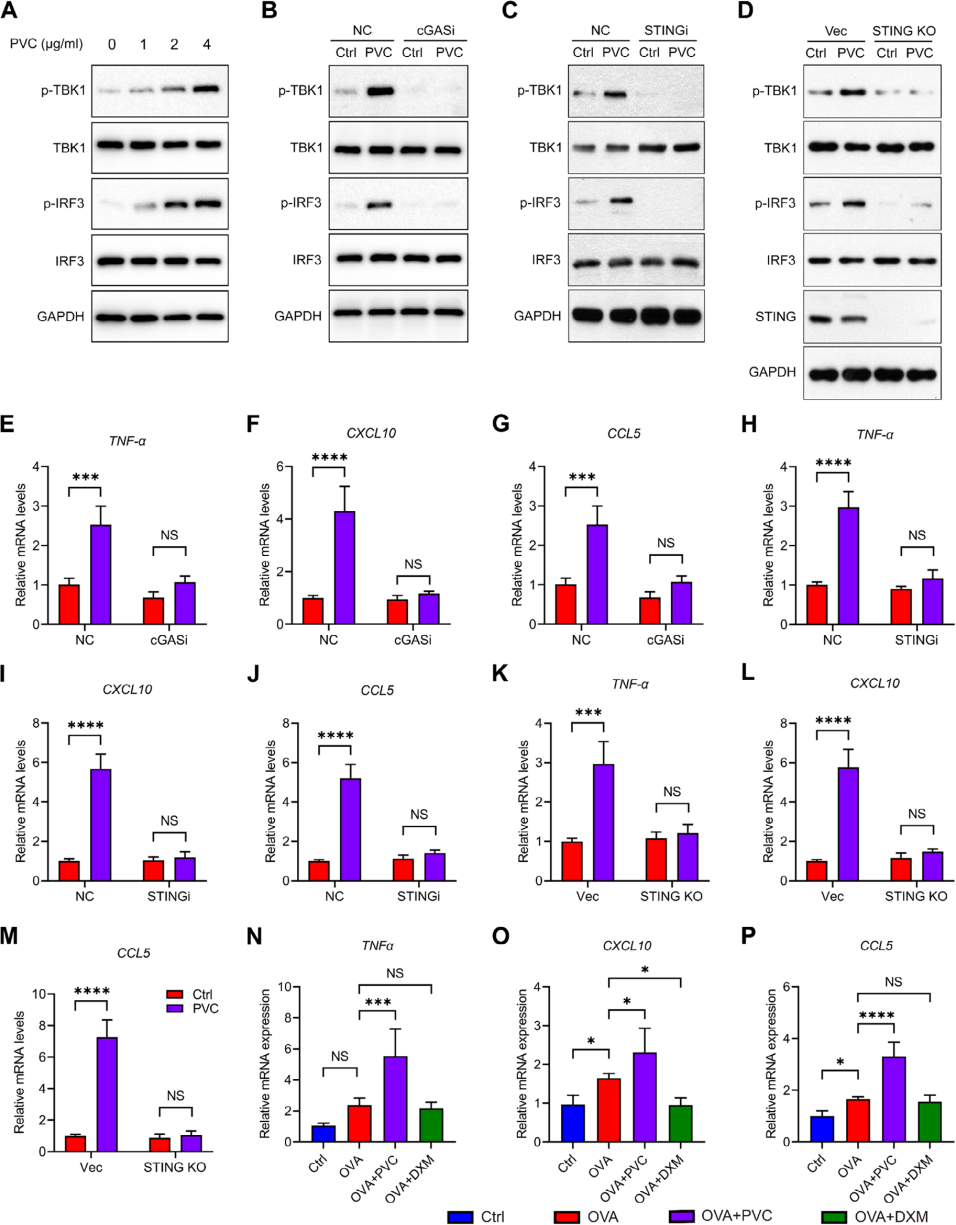
Activation of the cGAS‐STING pathway by PVC NPs. A) Western blotting showing dose‐dependent phosphorylation of TBK1 and IRF3 in THP‐1‐derived macrophages treated with PVC NPs for 48 h B–D) Effects of pharmacological inhibition or genetic ablation of the cGAS‐STING pathway on PVC NPs‐induced TBK1 and IRF3 phosphorylation: B) cGAS inhibitor (G140, 2 µm), C) STING inhibitor (H‐151, 10 nm), and D) STING KO THP‐1 cells. E–M) Relative mRNA expression of pro‐inflammatory cytokines (TNF‐α, CXCL10, and CCL5) in E–G) macrophages treated with PVC NPs ± cGAS inhibitor, H–J) macrophages treated with PVC NPs ± STING inhibitor, and K–M) STING KO cells treated with PVC NPs (*n* = 3). N–P) TNF‐α (N), CXCL10 (O), and CCL5 (P) mRNA levels in BALF cells from PVC NPs‐treated asthmatic mice (*n* = 5). Statistical analysis was performed via two‐way (E–M) or one‐way (N–P) ANOVA followed by Tukey's multiple comparison test. The data are presented as the mean ± SD; *p < 0.05, ***p < 0.001, ****p < 0.0001, NS, not significant. All the data are representative of three independent experiments.

Considering the important role of the cGAS‐STING pathway in immune responses and inflammation,^[^ ^43^, ^44^, ^45^, ^46^ ^]^ we investigated how PVC NPs may affect this signaling cascade. The results of this study demonstrate that PVC NPs exposure significantly activates key downstream molecules of the cGAS‐STING signaling pathway, as manifested by dose‐dependent increases in phosphorylated TBK1 (p‐TBK1) and phosphorylated IRF3 (p‐IRF3) protein expression levels (**Figure** **4**A). Further investigations revealed that prolonged exposure to PVC NPs resulted in a time‐dependent increase in the phosphorylation levels of TBK1 and IRF3 (Figure S6A, Supporting Information). Concurrently, RT‐qPCR analysis demonstrated time‐dependent upregulation of mRNA expression of cGAS‐STING pathway downstream inflammatory factors, including TNF‐α, CXCL10, and CCL5 (Figure S6B–D, Supporting Information). The present study demonstrates through intervention experiments with the cGAS‐specific inhibitor G140 (cGASi) and the STING inhibitor H‐151 (STINGi), combined with a STING knockout (STING KO) model, that PVC NPs exposure significantly activates the cGAS‐STING signaling pathway, as evidenced by markedly increased phosphorylation levels of TBK1 and IRF3, which were restored upon cGAS‐STING pathway inhibition (Figure 4B–D). Mechanistic investigations revealed that PVC NPs exposure upregulates the expression of the downstream inflammatory factors TNF‐α, CXCL10, and CCL5 via this pathway (Figure 3I–K), an effect that was reversed under conditions of cGAS inhibition, STING inhibition, or genetic knockout (Figure 4E–M). At the animal level, the expression of these inflammatory factors was further elevated in the BALF cells of the PVC NPs‐exposed asthmatic mice compared with those of the asthma‐only group (Figure 4N–P).

In conclusion, these results suggest that PVC NPs activate the cGAS‐STING pathway and promote the expression of downstream inflammatory mediators. This activation of cGAS‐STING may play a central role in the inflammatory responses triggered by PVC NPs exposure in immune cells.

### 2.5 PVC NPs Induce STING Pathway Activation in Macrophages through RNASEH1‐R‐Loop Signaling

**Figure 5:**
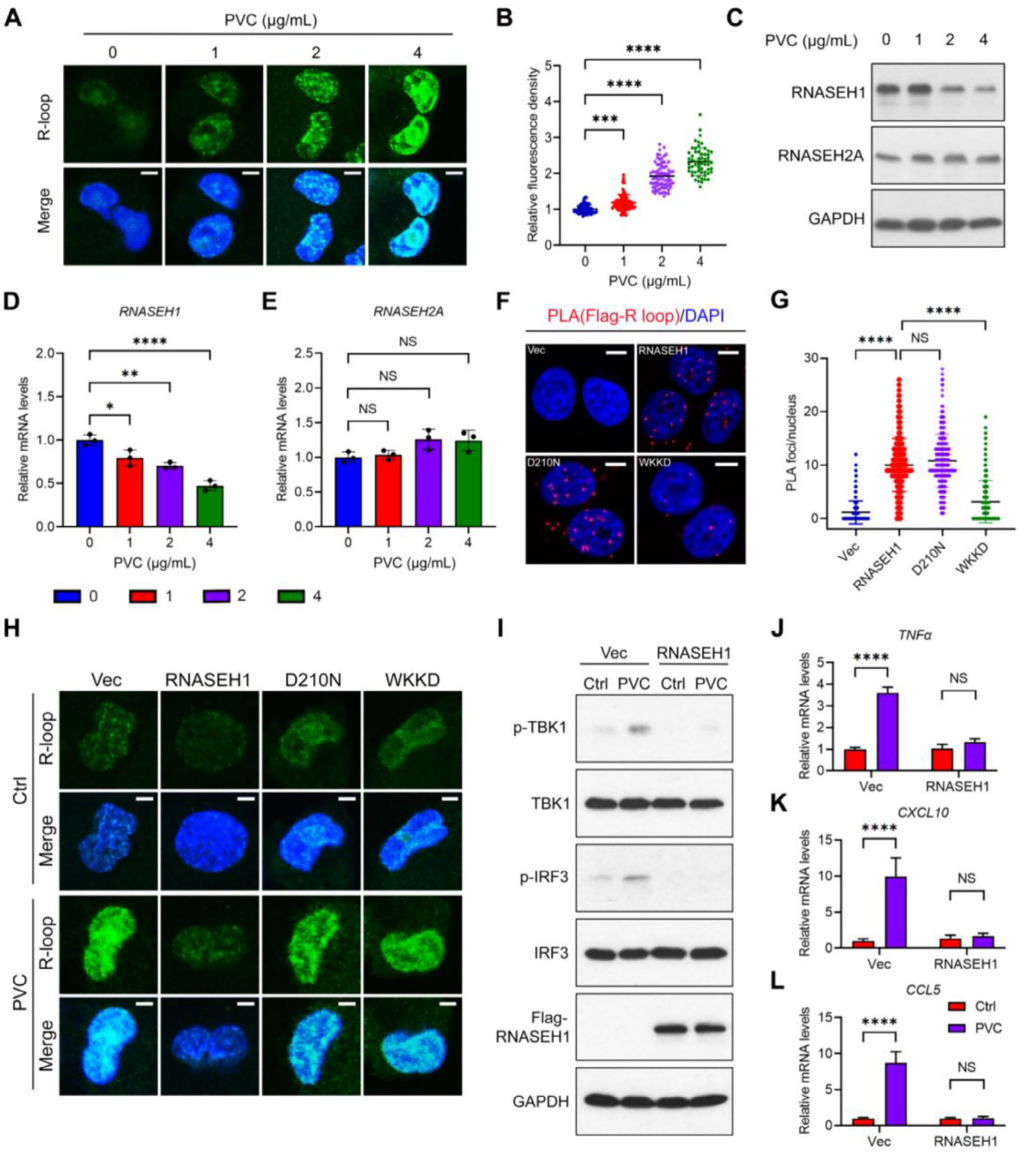
RNASEH1‐R‐loop signaling mediates PVC NPs‐induced STING pathway activation in macrophages. A) Representative composite fluorescence images showing the levels of R‐loops after treatment with different concentrations of PVC NPs. The R‐loop and nuclei were labeled with an anti‐DNA: RNA hybrid antibody (S9.6) (green) and DAPI (blue), respectively (scale bar = 5 µm). B) Quantitative analysis of the relative fluorescence intensity of R‐loops in the nucleus of cells treated with different concentrations of PVC NPs (*n* > 50 cells). C) The protein levels of RNASEH1 and RNASEH2A in cells exposed to different concentrations of PVC NPs were determined by western blotting. D,E) The relative mRNA levels of RNASEH1 (D) and RNASEH2A (E) were measured in THP‐1‐derived macrophages treated with different concentrations of PVC NPs for 48 h (*n* = 3). F) Representative R‐loop PLA fluorescence images demonstrating the RNASEH1‐R‐loop interaction (scale bar = 5 µm). G) Quantitative analysis of PLA foci in the nuclear compartment (*n* > 50 cells). H) Representative composite fluorescence images showing the levels of R‐loops after RNASEH1 overexpression following PVC NPs treatment (scale bar = 3 µm). I) The protein levels of TBK1, p‐TBK1, IRF3, p‐IRF3, and Flag‐RNASEH1 in cells exposed to or without PVC NPs were determined by western blotting. J–L) The relative mRNA levels of TNF‐α (J), CXCL10 (K), and CCL5 (L) were measured in THP‐1‐derived macrophages treated with PVC NPs after RNASEH1 overexpression (n = 3). Statistical analysis was performed using one‐way (B,D,E,G) or two‐way (J–L) ANOVA followed by Tukey's multiple comparison test. The data are presented as the mean ± SD; *p < 0.05, **p < 0.01, ***p < 0.001, ****p < 0.0001, NS, not significant. All the data are representative of three independent experiments.

In recent years, increasing evidence has suggested that activation of the cGAS‐STING pathway may be associated with the accumulation of R‐loops.^[^ ^31^, ^47^ ^]^ We investigated R‐loop accumulation via immunofluorescence staining with the R‐loop‐specific antibody S9.6. As a positive control, we used RNASEH1 inhibition, as RNASEH1 is a well‐known nuclease that specifically degrades R‐loops by disrupting RNA‐DNA hybrid structures.^[^ ^48^ ^]^ An RNASEH1 inhibitor leads to the accumulation of R‐loops in macrophages due to impaired R‐loop resolution (Figure S7A,B, Supporting Information). Similarly, exposure to PVC NPs caused a concentration‐dependent increase in R‐loop structures (**Figure** **5**A,B), indicating that PVC NPs exposure led to R‐loop accumulation in macrophages.

Given the crucial role of RNASEH enzymes, particularly RNASEH1 and RNASEH2, in resolving R‐loops,^[^ ^48^ ^]^ we hypothesized that exposure to PVC NPs might disrupt their activity and lead to R‐loop accumulation. We thus focused on the effects of PVC on these enzymes. Western blot analysis revealed a dose‐dependent decrease in RNASEH1 expression with increasing concentrations of PVC NPs, while RNASEH2A expression remained unaltered (Figure 5C). These findings suggest that PVC NPs exposure may downregulate RNASEH1 expression, thereby impairing R‐loop resolution. Consistent with the protein‐level results, RT‐qPCR analysis confirmed reduced RNASEH1 expression in macrophages following PVC NPs exposure (Figure 5D,E). A proximity ligation assay (PLA) further validated the specific interaction between RNASEH1 and R‐loop. Notably, while the catalytically deficient RNASEH1 mutant D210N retained R‐loop binding capacity, the non‐binding catalytic RNASEH1 mutant WKKD (W43A, K59A, K60A, and D210N) barely interacted with R‐loop (Figure 5F,G). Furthermore, immunofluorescence experiments revealed that the overexpression of RNASEH1, but not the D210N mutant or WKKD mutant, eliminated the increase in the R‐loop caused by PVC NPs exposure (Figure 5H), further confirming the important role of this nuclease in PVC NPs‐induced R‐loop accumulation. Moreover, PVC NPs exposure triggered the phosphorylation of the STING downstream signaling proteins TBK1 and IRF3, an effect that was reversed by RNASEH1 overexpression, which highlights the dependence of PVC NPs‐induced STING pathway activation on RNASEH1 (Figure 5I). Consistently, RNASEH1 overexpression abolished the increased levels of the downstream inflammatory factors TNF‐α, CXCL10, and CCL5 in the cGAS‐STING pathway (Figure 5J–L). These results further suggest that PVC NPs may promote the abnormal accumulation of R‐loops and activate the downstream cGAS‐STING signaling pathway by inhibiting RNASEH1 expression.

### 2.6 STING KO Alleviates PVC NPs‐Induced Inflammation in Allergic Asthma

**Figure 6:**
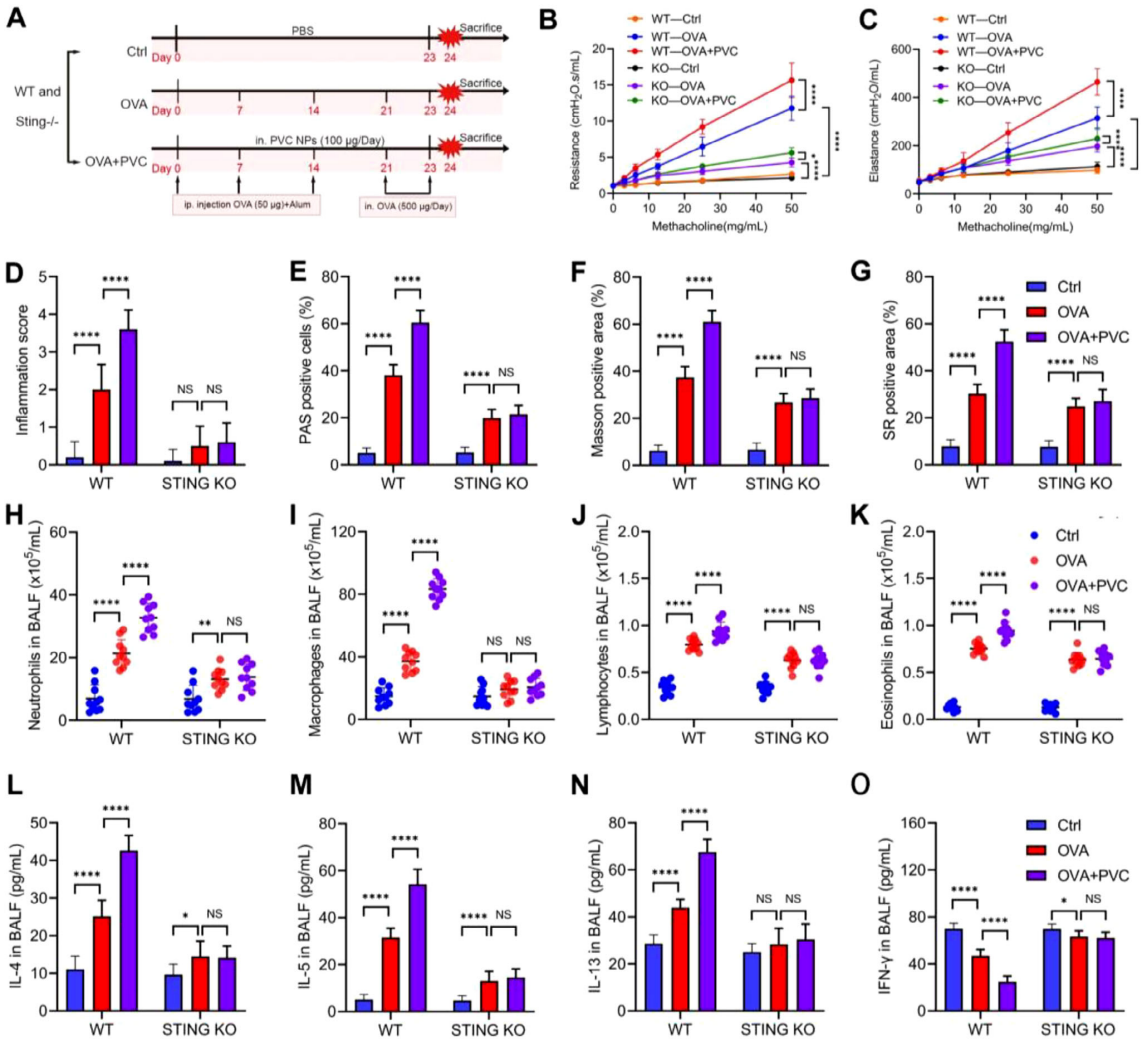
STING KO reduces PVC NPs‐triggered inflammation in asthmatic models. A) OVA‐induced asthma model and PVC NPs exposure in STING KO or wild‐type mice. B,C) Changes in airway resistance (Rn) and airway elastance (Ers) were measured in anesthetized animals in response to inhaled increasing concentrations of methacholine (0–50 mg mL^−1^) 24 h after the last OVA stimulation (*n* = 5). D) Quantitative inflammation scores of lung tissue via H&E staining (*n* = 10). E) Quantification of PAS‐positive cells in lung tissue via PAS staining (*n* = 10). F) Quantification of the positive area of Masson‐stained lung tissue (*n* = 10). G) Quantitative SR‐positive area of lung tissue subjected to SR staining (*n* = 10). H–K) Quantitative analysis of the number of neutrophils (H), macrophages (I), lymphocytes (J), and eosinophils (K) in the BALF of each group of mice (*n* = 10). L–O) Quantitative analysis of the IL‐4 (L), IL‐5 (M), IL‐13 (N), and IFN‐γ (O) concentrations in the BALF of each group of mice via ELISA (*n *= 10). Statistical analysis was performed using two‐way ANOVA followed by Tukey's multiple comparison test. The data are presented as the mean ± SD; *p < 0.05, **p < 0.01, ****p < 0.0001, NS, not significant.

To further confirm whether PVC NPs affect the inflammatory response in allergic asthma through the STING pathway, we constructed an OVA‐induced allergic asthma mouse model in both WT and STING KO mice and exposed them to PVC NPs (**Figure** **6**A; Figure S8A, Supporting Information). To assess the impact on airway function, we measured the total respiratory system resistance and elasticity of each group of mice after the final OVA challenge. The resistance and elasticity in the PVC NPs exposure group were significantly higher than those in the control group, and this upregulation was reversed after STING KO (Figure 6B,C). Similarly, we stained the lung tissues of each group of mice using methods such as H&E, PAS, Masson, and SR. Overall, compared with the control treatment, PVC NPs exposure resulted in increased inflammatory cell infiltration and enhanced inflammation (Figure 6D), with a higher number of PAS‐positive cells (Figure 6E). Additionally, Masson and SR staining revealed increased collagen deposition in the lung tissues of the mice exposed to PVC NPs (Figure 6F,G), and these exacerbated responses were reversed by STING gene knockout. As mentioned earlier, we also assessed changes in inflammatory cells in mouse BALF. As expected, STING KO alleviated the increase in various inflammatory cells, including macrophages, induced by PVC NPs exposure (Figure 6H–K; Figure S8B, Supporting Information). Finally, ELISA analysis of various cytokines in the BALF revealed that Th2‐type cytokines, such as IL‐4, IL‐5, and IL‐13, were significantly increased after PVC NPs exposure, whereas the Th1‐type cytokine IFN‐γ was significantly decreased (Figure 6L–O). No significant differences were observed in the STING KO mice. We assessed baseline inflammation in unchallenged WT and STING KO mice. STING KO mice exhibited slightly reduced basal inflammation, consistent with its immunomodulatory role.^[^ ^49^ ^]^ Importantly, the exacerbation of inflammation by PVC NPs was only observed in WT mice and not in STING KO mice, indicating a PVC NPs‐specific and STING‐dependent mechanism. These results suggest that the STING pathway participates in the process by which PVC NPs exacerbate OVA‐induced pulmonary inflammation in allergic asthma mice. Consistently, ELISA analysis of murine serum samples revealed that STING KO significantly reversed the PVC NPs exposure‐induced elevation in both total IgE and sIgE levels (Figure S8C,D, Supporting Information).

## 3 Discussion

PVC NPs have garnered significant attention because of their widespread use in products such as packaging, construction materials, and medical devices. Due to their durability and resistance to degradation, PVC particles accumulate rapidly in the environment, posing an increasing health risk through human exposure.^[^ ^7^, ^11^, ^12^ ^]^ In recent years, studies have highlighted the potential cytotoxic effects of NPs, especially PVC NPs, which can induce a variety of cellular responses, such as oxidative stress, mitochondrial damage, and apoptosis.^[^ ^7^, ^21^, ^22^, ^23^, ^24^, ^50^, ^51^, ^52^, ^53^, ^54^ ^]^ Animal models have shown that NPs exposure is associated with adverse effects, including immune dysfunction and metabolic changes.^[^ ^55^, ^56^, ^57^ ^]^ Among different NPs, PVC NPs are known to exert stronger cytotoxic and inflammatory effects than other common polymer types, reinforcing their high‐risk potential in respiratory exposure contexts.^[^ ^50^, ^51^, ^52^, ^53^, ^54^ ^]^ The small size and light weight of PVC NPs enable them to deeply penetrate and accumulate in the lungs.^[^ ^14^, ^15^ ^]^ As the primary^[^ ^50^, ^51^, ^52^, ^53^, ^54^ ^]^ interface between the external environment and the body, the lung is particularly vulnerable to nanoparticle exposure, making it an important target for investigating the respiratory impacts of PVC NPs. However, despite the increasing body of research, the specific molecular mechanisms by which respiratory exposure to micro‐ and nanoplastics triggers inflammatory responses remain largely unclear. In this study, we identified R‐loop accumulation as a key cellular event in PVC NPs‐induced inflammation in macrophages, demonstrating how R‐loop accumulation activates the cGAS‐STING pathway, thus offering new insights into the pathogenesis of asthma. Our findings provide a better understanding of the molecular mechanisms involved in PVC NPs toxicity and highlight potential targets for mitigating the health risks associated with microplastic pollution.

Asthma is a common inflammatory respiratory disease with a complex pathogenesis influenced by genetic predispositions, environmental exposures, and their interactions.^[^ ^1^, ^2^, ^3^ ^]^ Our findings suggest that PVC NPs may exacerbate the inflammatory response in asthma by promoting R‐loop formation in alveolar macrophages. This newly identified mechanism provides additional insights into the pathophysiology of asthma and underscores the potential risks associated with environmental exposure to NPs. To further investigate the role of the cGAS‐STING pathway, we employed STING KO mice, which presented significantly reduced inflammatory responses upon PVC NPs exposure. These results confirm the critical role of STING activation in mediating the inflammatory effects of PVC NPs and highlight the cGAS‐STING pathway as a potential target for therapeutic intervention. Together, our findings emphasize the importance of limiting PVC NPs exposure as a preventive measure and suggest that targeting STING may hold promise for mitigating asthma exacerbations driven by environmental pollutants.

PVC NPs exposure resulted in clear immune activation, including elevated Th2‐type cytokines (IL‐4, IL‐5, and IL‐13) and reduced IFN‐γ levels, along with a shift toward a pro‐inflammatory macrophage gene profile. THP‐1‐derived macrophages exposed to BALF from PVC NPs‐exposed mice also showed increased migration and increased expression of chemokines, such as CXCL10 and CCL5, as well as inflammatory mediators, including TNF‐α and iNOS. These results suggest that PVC NPs exposure not only activates macrophages but also promotes their accumulation in inflamed airways. Similar macrophage responses have been reported in nanoparticle‐induced inflammation models, where particle exposure enhances both immune cell infiltration and cytokine production through innate immune signaling.^[^ ^58^, ^59^, ^60^ ^]^ These immune signatures are consistent with previous studies showing that STING activation in alveolar macrophages promotes airway hyperresponsiveness and Th2 inflammation^[^ ^38^ ^]^ and that STING agonists exacerbate house dust mite‐induced asthma by inducing neutrophilic infiltration and increasing IFN‐γ and IL‐6 production.^[^ ^49^ ^]^ Together, our data suggest that PVC NPs function not only as particulate irritants but also as immunostimulatory agents capable of reshaping pulmonary immune dynamics.

Mechanistically, we identified R‐loop accumulation as the central event linking PVC NPs exposure to innate immune activation. PVC NPs exposure led to significant downregulation of RNASEH1, an essential enzyme for resolving RNA‐DNA hybrids, resulting in R‐loop accumulation in alveolar macrophages. This abnormal buildup of R‐loops subsequently activated the cGAS‐STING pathway, triggering downstream inflammatory signaling via the IRF3/type I IFN and NF‐κB axes. These findings were supported by experiments using both pharmacological inhibition and STING KO models, which significantly reduced airway inflammation following PVC NPs challenge. R‐loop structures are typically formed during transcription and play physiological roles in gene regulation and genome stability^[^ ^27^ ^]^ but their pathological accumulation has been linked to DNA damage and inflammatory signaling.^[^ ^30^, ^31^, ^32^, ^36^ ^]^ While previous studies have associated R‐loop dysregulation with autoimmunity and senescence, our study is the first to establish a direct connection between R‐loop accumulation and asthma pathogenesis. This R‐loop‐STING axis provides a novel mechanistic framework for understanding how environmental particulates engage nuclear stress and cytosolic immune sensing to drive chronic inflammation in the lung.

These mechanistic insights also point toward new therapeutic opportunities. Targeting the cGAS‐STING pathway, a central mediator of innate immune inflammation, may represent a novel strategy for mitigating pollutant‐aggravated asthma. Small‐molecule STING inhibitors such as H‐151 have demonstrated efficacy in various preclinical models of lung disease, including LPS‐induced acute lung injury,^[^ ^61^ ^]^ radiation‐induced pneumonitis,^[^ ^62^ ^]^ and steroid‐resistant asthma.^[^ ^63^ ^]^ Moreover, restoring RNASEH1 function, either through mRNA delivery or small‐molecule activators, could suppress upstream R‐loop accumulation and potentially attenuate inflammation at its source. Although these approaches are still exploratory, they offer promising avenues for therapeutic intervention in asthma and other pollution‐exacerbated inflammatory conditions.

While our study was designed to simulate high‐end human exposure scenarios, we acknowledge that real‐world PVC NPs exposure is generally chronic, relatively low in concentration, and occurs alongside other airborne pollutants such as metals. The selected dose in the murine asthma model was derived from inhalation estimates and scaled by body surface area,^[^ ^64^, ^65^ ^]^ modeling an acute, high‐exposure event. Future work should explore long‐term, low‐dose regimens and include complex indoor aerosol matrices to better reflect real‐world exposure dynamics and cumulative risk. Additionally, environmental nanoplastics exhibit high heterogeneity in size, shape, and polymer composition. Although we focused on spherical PVC NPs (≈50 nm), recent comparative studies have shown that nanoplastics of similar size but different polymer types (e.g., PET vs PS) display markedly different cytotoxicity, oxidative stress responses, and gene expression patterns in exposed cells.^[^ ^66^ ^]^ These findings reinforce the need for future mechanistic research using diverse polymer types (e.g., PE, PS, PET) and a range of sizes and shapes to fully understand the differential immune and inflammatory effects of environmental nanoplastics. Furthermore, while our findings were robust in murine and THP‐1 models, validation using primary human macrophages and clinical samples (e.g., BALF from exposed asthma patients) will be crucial for translational relevance. Finally, although we profiled major innate and adaptive immune cell populations, future studies should investigate T‐cell subsets, eosinophils, and the broader immunological landscape associated with chronic microplastic inhalation more comprehensively.

Together, our findings present a new framework for understanding how PVC NPs contribute to inflammatory diseases such as asthma. By linking PVC NPs exposure to R‐loop accumulation and cGAS‐STING pathway activation, we identified molecular targets that could inform therapeutic interventions. In addition to asthma, this research highlights the broader implications of NPs exposure in inflammatory pathologies, suggesting potential strategies to reduce pollutant‐driven health impacts.

## 4 Experimental Section

### Reagents and Antibodies

PVC NPs (50 nm) and fluorescently labeled PVC NPs (50 nm) were purchased from Jiangsu Zhichuan Technology. The PVC NPs solution used in the experiment was composed of PBS. Ovalbumin (OVA) (# A5503) was purchased from Sigma‐Aldrich. The aluminum hydroxide adjuvant (# 77 161) was purchased from Thermo Fisher Scientific. Anti‐IRF3 (# 11 904), anti‐p‐IRF3 (# 29 047), anti‐TBK1 (# 3504), anti‐p‐TBK1 (# 5483), and anti‐GAPDH (# 2118) antibodies were obtained from Cell Signaling Technology. Anti‐RNASEH1 (# sc‐376326) was obtained from Santa Cruz Biotechnology. Anti‐RNASEH2A (# ab92876) was ordered from Abcam. An anti‐DNA‐RNA hybrid antibody (S9.6) (# GK60001) was purchased from AntibodySystem. The anti‐FLAG antibody (#66008‐4‐lg) was obtained from Proteintech. Mouse IL4 (# M4000B), mouse IL‐5 (# M5000), and mouse IFN‐γ (# MIF00) ELISA kits were obtained from R&D Systems. Mouse IL13 (# ECM010) and mouse IgE (# EMIGHE) ELISA kits were purchased from Thermo Fisher Scientific. A mouse OVA‐specific ELISA kit (# 439 807) was purchased from BioLegend. APC‐Cy7 Rat Anti‐Mouse CD45 ^(30‐F11)^ (#557 659), BUV395 Hamster Anti‐Mouse CD3e (145‐2C11) (#563 565), BV421 Rat Anti‐Mouse CD4 (GK1.5) (#502 891), Brilliant Violet 605 Anti‐Mouse CD8a (#100 744), FITC Rat Anti‐CD11b (M1/70) (#557 396), PE Rat Anti‐Mouse F4/80 (T45‐2342) (#565 410), Alexa Fluor 700 Rat Anti‐Mouse Ly‐6G (1A8) (#561 236), Brilliant Violet 650 Anti‐Mouse Ly‐6C (#128 049), PE‐Cy7 Hamster Anti‐Mouse CD11c (HL3) (#558 079), V500 Rat Anti‐Mouse I‐A/I‐E (M5/114.15.2) (#562 366), and 7‐AAD (#559 925) were obtained from BD Pharmingen. Anti‐mouse CD19 (eBio1D3) BV786 (#417‐0193‐82) was purchased from Thermo Fisher Scientific.

### Cell Culture

The human cell lines THP‐1 (# TIB‐202) and 293T (# CRL‐3216) were obtained from American Type Culture Collection (ATCC) and cultured in RPMI‐1640 medium or Dulbecco's modified Eagle's medium (DMEM) supplemented with 10% FBS (Gibco, # 10099141C), 100 U mL^−1^ penicillin, and 100 µg mL^−1^ streptomycin. All the cells were maintained at 37 °C in a humid 5% CO_2_ incubator. To generate macrophage‐like cells, the seeded THP‐1 cells were treated with 1 µm PMA (Beyotime, # S1819), a concentration selected based on the manufacturer's instructions and previous experience,^[^ ^67^, ^68^ ^]^ for 48 h. To inhibit cGAS activation, the cGAS‐specific inhibitor G140 (MedChemExpress, #HY‐133916) was administered at a concentration of 2 µm. The STING inhibitor H‐151 (MedChemExpress, # HY‐112693) was used to block STING activation at a concentration of 10 nm. The RNASEH1 plasmid (Addgene, #65 782), catalytically deficient RNASEH1 mutant D210N plasmid (Addgene, #111 904), or non‐binding catalytic RNASEH1 mutant WKKD plasmid (a RNASEH1 plasmid contains W43A, K59A, K60A, and D210N mutant, which lost its binding ability to RNA/DNA hybrids) (Addgene, #111 904) was transfected to generate THP‐1 RNASEH1 or mutant ‐overexpressing cells. STING was knocked out via the CRISPR‐Cas9 system according to a protocol described previously.^[^ ^69^ ^]^ Briefly, DNA primers for the STING gRNA and reverse complement sequence (forward primer, 5′‐ CACCGGCGGGCCGACCGCATTTGGG‐3′; reverse primer, 5′‐ AAACCCCAAATGCGGTCGGCCCGCC ‐3′) were synthesized and ligated into LentiCRISPR v2 (Addgene #52 961). Lentiviruses were generated in HEK293T cells. THP‐1 cells were infected with lentivirus and then selected with puromycin before experiments.

### Characterization of PVC NPs

The morphology of the 50 nm PVC NPs was characterized by SEM (JEOL JSM‐IT800, Japan) and transmission electron microscopy (TEM, JEOL JEM‐2100plus, Japan). The hydrodynamic diameter and zeta potential were measured via a nanoparticle analyzer (Malvern Panalytical Zetasizer Ultra, UK). To evaluate material stability, the PVC NPs were suspended in PBS, incubated at 37 °C for 72 h, and then centrifuged. The collected samples were vacuum‐dried and analyzed via Fourier transform infrared spectroscopy (FT‐IR, Thermo Fisher Nicolet iS50, USA) alongside fresh PVC NPs, with a scanning range of 500‐4000 cm^−1^.

### Animal Work

Specific pathogen‐free (SPF) BALB/c mice (6—8 weeks old) obtained from GemPharmatech (China) were randomly divided into six experimental groups (*n* = 10 per group): control (Ctrl, PBS‐treated), OVA‐sensitized asthma model (OVA), OVA with low‐dose PVC NPs (OVA+PVC Low, 10 µg day^−1^), OVA with medium‐dose PVC nanoparticles (OVA+PVC Medium, 50 µg day^−1^), OVA with high‐dose PVC nanoparticles (OVA+PVC High, 100 µg day^−1^), and OVA with dexamethasone treatment (OVA+DXM). Based on established experimental protocols,^[^ ^70^, ^71^ ^]^ the asthma model was established by intraperitoneal sensitization with 20 µg of OVA plus 100 µL of aluminum hydroxide suspension (40 mg mL^−1^) and 100 µL of PBS on days 0, 7, and 14, followed by intranasal challenge with 25 mg mL^−1^ OVA (500 µg per challenge in 50 µL of PBS, administered as 20 µL aliquots) from days 21 to 23. Concurrently, the PVC NPs‐exposed groups received daily intranasal administration of PVC NPs at the indicated doses (10, 50, or 100 µg in 20 µL of PBS). PVC NPs doses were selected based on estimated human exposure levels, interspecies scaling, and validation in dose‐response pilot studies.^[^ ^64^, ^65^, ^72^, ^73^, ^74^ ^]^ Characteristic asthma behaviors, including restlessness and nose scratching, were observed during the challenges. The OVA+DXM group received daily intraperitoneal dexamethasone (0.2 mg mL^−1^, 2 mg kg^−1^) from day 15 onward.^[^ ^75^ ^]^ The OVA+DXM group was included in the experimental design solely as a positive control for therapeutic intervention, to confirm that the OVA‐induced asthma model was successfully established and remained pharmacologically responsive. All the mice were euthanized within 24 h after the final challenge, with blood samples collected via the orbital sinus and serum separated by centrifugation for storage at −80 °C.

STING knockout (KO) and wild‐type (WT) C57BL/6 mice, aged 6—8 weeks, were purchased from GemPharmatech, China. To validate the genotype of the STING KO mice, genomic DNA was extracted from the toe tissue, and PCR amplification was performed using 2X Accurate Taq Master Mix (Accurate Biology, # AG11007). The primers for the STING KO allele: Forward (5′‐ACCTGATGGGAGGTATCTACCGG‐3′) and Reverse (5′‐CCAGCAACTAGCATCAGAACCTCC‐3′). The primers for the wild‐type allele were: Forward (5′‐GGTGCCTGACAACCTGAGTGTAG') and Reverse (5′‐CCTCAATGCTCTCATAGCCTTCAC‐3′). The PCR products were analyzed by electrophoresis on a 1.5% agarose gel. The STING KO and WT mice were randomly divided into two groups, with five mice in each group, and sensitized and challenged via the same OVA protocol described above. The mice in the PVC NPs exposure group received 100 µg of nasal drops daily, whereas those in the control group were administered only PBS. The animal experiments were approved by the Animal Protection and Utilization Committee of Hangzhou Institute of Medicine, Chinese Academy of Sciences (No. 2022R0012).

### Airway Hyperresponsiveness (AHR) Detection

Methacholine challenge is a well‐established test of AHR, a hallmark of asthma pathophysiology.^[^ ^76^ ^]^ After the final OVA stimulation treatment for 24 h, the mice were anesthetized and fixed on the experimental platform. Following disinfection with alcohol, the trachea was exposed, and tracheal intubation was performed. The cotton thread was used to secure the catheter, connect it to the ventilator, and gradually aerosolize methacholine into the airways with increasing concentration. The resistance and elastance of the respiratory system were measured and analyzed using the FlexiVent system (Scireq, Montreal, QC, Canada).

### Bronchoalveolar Lavage Fluid (BALF) and Infiltrated Cell Counts

After airway hyperresponsiveness was assessed, tracheal intubation was performed for BALF. The bronchi were lavaged three times with cold phosphate‐buffered saline (PBS) (1 mL per lavage). The collected BALF was subsequently centrifuged to pellet the cells, and the supernatant was stored at −80 °C until it was used for cytokine analysis. The cell pellets were resuspended in PBS, and the total quantity of inflammatory cells was determined via a hemocytometer. For leucocyte classification and counting, a smear of the cell pellet from the BALF was prepared and examined by Wright–Giemsa staining (Baso, # BA4017).

### ELISA

BALF and blood samples were collected from the mice. The BALF was centrifuged to separate the supernatant, and the blood samples were centrifuged to obtain the serum. Both the BALF supernatant and the serum were stored at −80 °C until further analysis. The concentrations of IL‐4, IL‐5, IL‐13, and IFN‐γ in the BALF were measured via commercial ELISA kits according to the manufacturer's instructions. Similarly, total IgE and specific IgE (sIgE) levels in the serum were quantified via commercially available ELISA kits. Three technical replicates of ELISA were performed for each mouse.

### Lung Histopathology

After the experiment, the mouse lung tissues were collected and fixed with 4% paraformaldehyde for 48 h. The samples were then rinsed with distilled water and dehydrated through a series of ethanol gradients. The tissues were treated with xylene for ≈20 min before being embedded in paraffin. The paraffin‐embedded sections were subjected to hematoxylin and eosin (H&E), periodic acid‐Schiff (PAS), Masson's trichrome, and Sirius Red (SR) staining. The assessment of pulmonary inflammation was conducted in a blinded manner using H&E staining with an established inflammatory scoring system:^[^ ^77^ ^]^ 0, indicating no detectable inflammation; 1, representing occasional inflammatory cell infiltration; 2, denoting 1‐3 layers of inflammatory cells surrounding most bronchi or blood vessels; 3, indicating 4‐5 layers of inflammatory cell infiltration around the majority of bronchi or vessels; and 4, representing more than 5 layers of inflammatory cell infiltration around most bronchi or blood vessels. To quantify goblet cell hyperplasia, PAS staining was performed on tissue sections from each mouse, with PAS‐positive substances appearing red to purple and nuclei stained light blue, and the percentage of PAS‐positive cells in the epithelial area was calculated using ImageJ software. Following established methodologies,^[^ ^78^ ^]^ Masson's trichrome staining was employed to evaluate collagen deposition in airways and lung parenchyma, with the collagen‐stained blue areas quantified as a percentage of the total examined area to determine the collagen fiber content. Similarly, SR staining was used to assess collagen fiber deposition in lung tissue, where the percentage of red‐stained positive areas was quantified under normal light microscopy to evaluate the collagen content, whereas under polarized light, specifically stained collagen areas (type I collagen appearing orange‐red and type III collagen appearing green) were examined to determine the content of different collagen fiber types.

### Behavioral Tests

Previous studies have shown that asthma leads to behavioral symptoms such as fatigue, lethargy, and reduced exercise endurance, which are significantly positively correlated with disease severity.^[^ ^79^, ^80^ ^]^ Additionally, repeated nose‐rubbing behavior is a typical ethological feature of OVA‐induced allergic asthma.^[^ ^42^ ^]^ Following the final asthma challenge, behavioral tests were conducted on the mice from each experimental group. The open field test was conducted in a room with indoor lighting and a quiet environment. Each mouse was placed in the center of an open box, and before the behavioral test, each mouse was allowed to adapt to the open box environment for 10 min. After each mouse was subjected to the experiment, it was immediately removed, and its feces and urine were removed. Finally, the bottom and sidewalls of the open box were cleaned with a cloth soaked in 70% alcohol, and the next mouse behavioral test was conducted only after the alcohol had completely evaporated. The duration of the open field test for each mouse was set to 10 min. The total route and distance the mouse moved within 10 min were analyzed via Tracker software (Supermaze). Moreover, the cumulative stationary time, the number of times the mice stood upright on their hind limbs, and the frequency with which the nose touched their forepaws (retouching) within a ten‐minute period for each group of mice were statistically analyzed.

### Flow Cytometry Analysis

Flow cytometry was employed to analyze the distribution of immune cell subsets in the BALF. Briefly, BALF was centrifuged at 300 g for 10 min at 4 °C to prepare single‐cell suspensions. The cells were stained with fluorescent antibodies in PBS/1% FBS for 30 min, using the following pre‐titrated antibodies: FITC‐anti‐CD11b and PE‐anti‐F4/80 for macrophages; PE‐Cy7‐anti‐CD11b and AF700‐anti‐Ly6G for neutrophils; PE‐Cy7‐anti‐CD11c and V500‐anti‐MHC II for dendritic cells; FITC‐anti‐CD11b and BV650‐anti‐Ly6C for monocytes; and BUV395‐anti‐CD3 and BV421‐anti‐CD4 and BV605‐anti‐CD8 for T cells, and 7‐AAD was added prior to acquisition to exclude dead cells. The stained cells were analyzed on a SONY SP6800Z flow cytometer, and the data were processed via FlowJo software.

### Cell Counting Kit‐8 (CCK8) Assay

The effect of PVC NPs on the proliferation of THP‐1‐derived macrophages was tested using a CCK‐8 assay kit according to the manufacturer's instructions. THP‐1 cells were seeded in a 96‐well plate, and after PMA‐induced adherence, different concentrations of PVC NPs (four replicates per concentration) were added and incubated for 48 h. Then, 10 µL of CCK‐8 solution was added to each well, followed by incubation at 37 °C for 2 h. The absorbance was measured at 450 nm via a microplate reader (Tecan, Switzerland).

### Migration Assay

THP‐1 cells were seeded at a density of 2 × 10⁴ cells well^−1^ in the upper chamber of a 24‐well transwell plate and incubated with 1 µm PMA for 48 h to induce their differentiation into macrophages. After induction, 300 µL of culture medium mixed with 300 µL of BALF supernatant from the mice in each group was added to the lower chamber and incubated for 24 h. Following treatment, the medium was aspirated, and the cells were washed three times with PBS. The entire chamber was fixed with 4% paraformaldehyde for 30 min and then stained with 0.1% crystal violet for 30 min. After being washed and air‐dried, the cells on the lower surface of the membrane were photographed under a microscope. Each experiment was independently repeated three times, and the number of cells was determined via ImageJ software.

### Western Blotting

The experiments were performed as previously described.^[^ ^69^, ^81^ ^]^ After the cells were lysed with RIPA lysis buffer (Beyotime, # P0013B) and the supernatant was collected, the protein concentration was determined via a BCA protein assay kit (Beyotime, # P0010). The samples were then boiled at 100 °C for denaturation and subjected to sodium dodecyl sulfate‐polyacrylamide gel electrophoresis. The separated proteins were transferred onto a polyvinylidene fluoride (PVDF) membrane, which was then blocked with 5% nonfat milk at room temperature for 2 h. After washing, the membrane was incubated with the primary antibody at 4 °C overnight, followed by washing with PBS and incubation with the appropriate secondary antibody for 2 h. The membrane was then developed via a chemiluminescence detection kit (Beyotime, # P0018AS). The experiment was performed with three independent replicates.

### Reverse Transcription Quantitative PCR (RT‑qPCR)

Total RNA was extracted from the indicated cells via TRIzol reagent (Takara, #9109) and then reverse‐transcribed into cDNA via a PrimeScript RT Reagent Kit (Takara, #RR036A). qPCR was performed on a CFX96 Real‐Time PCR Detection System (Bio‐Rad, USA) with a Taq Pro Universal SYBR qPCR Master Mix Kit (Vazyme, # Q712‐02) following the manufacturer's guidelines. GAPDH was used as an internal reference, and the data were analyzed via the 2^−ΔΔCt^ method. Each assay was repeated at least three times. The sequences of the primers are listed in **Table** **1**. A minimum of three biological and technical replicates were performed.

**Table 1:** Sequences of primers for RT‐qPCR.

Gene	Primers	Sequence
RNASEH1 (human)	Forward	5’‐TCTTCCGATTGTTTAGCTCCTTC‐3’
	Reverse	5’‐TAACTGGGTTCAAGGTTGGAAG‐3’
RNASEH2A (human)	Forward	5’‐TAATGCCTCCTTCCCGCTTA‐3’
	Reverse	5’‐GACACCTGACCCAGGAATTGA‐3’
iNOs (human)	Forward	5’‐AGCCTGTGAGACGTTTGATGT‐3’
	Reverse	5’‐TGTAGATTCTGCCGAGATTTGA‐3’
TNF‐α (human)	Forward	5’‐TTGAGGGTTTGCTACAACATGGG‐3’
	Reverse	5’‐GCTGCACTTTGGAGTGATCG‐3’
CXCL10 (human)	Forward	5’‐TCTGAATCCAGAATCGAAGG‐3’
	Reverse	5’‐CTCTGTGTGGTCCATCCTTG‐3’
CD163 (human)	Forward	5’‐CGGCTGCCTCCACCTCTAAGT‐3’
	Reverse	5’‐ATGAAGATGCTGGCGTGACA‐3’
CD206 (human)	Forward	5’‐ACCTCACAAGTATCCACACCATC‐3’
	Reverse	5’‐CTTTCATCACCACACAATCCTC‐3’
Arg‐1 (human)	Forward	5’‐GGCTGGTCTGCTTGAGAAAC‐3’
	Reverse	5’‐ATTGCCAAACTGTGGTCTCC‐3’
CCL5 (human)	Forward	5’‐ATATGGCTCGGACACCACTC‐3’
	Reverse	5’‐TCCTTCGAGTGACAAACACG‐3’
CCL8 (human)	Forward	5’‐AGCTGTCCCTGTCAGCCCAGA‐3’
	Reverse	5’‐AGCAGCAGGTGACTGGAGCCT‐3’
CXCL11 (human)	Forward	5’‐ATGAGTGTGAAGGGCATGGC‐3’
	Reverse	5’‐TCACTGCTTTTACCCCAGGG‐3’
IFIT3 (human)	Forward	5’‐CAGAACTGCAGGGAAACAGC‐3’
	Reverse	5’‐TGAATAAGTTCCAGGTGAAATGGC‐3’
TLR7 (human)	Forward	5’‐TCTTTGGGTTTCGATGGTTTCC‐3’
	Reverse	5’‐GCAGTCCACGATCACATGGG‐3’
IFIT1 (human)	Forward	5’‐TTGATGACGATGAAATGCCTGA‐3’
	Reverse	5’‐ CAGGTCACCAGACTCCTCAC‐3’
IL10 (human)	Forward	5’‐GCCTAACATGCTTCGAGATC‐3’
	Reverse	5’‐TGATGTCTGGGTCTTGGTTC‐3’
MMP1 (human)	Forward	5’‐CTGCTGCTGCTGTTCTGGGGT‐3’
	Reverse	5’‐CCACTGGGCCACTATTTCTCCGCT‐3’
IL24 (human)	Forward	5’‐ACCCACAGCTATGCCTCTGATTG‐3’
	Reverse	5’‐TGTTAAATTGGCGAAAGCAGCTC‐3’
PPBP (human)	Forward	5’‐CGTTGTTCCCTCCTGGCTCT‐3’
	Reverse	5’‐GGACGATGTAGGTCTGAGTC‐3’
AOX1 (human)	Forward	5’‐TGTCGATCCTGAAACAATGCTG‐3’
	Reverse	5’‐GGTGATGGGGTTGTATCGTGA‐3’
LOXL4 (human)	Forward	5’‐CCGCTGCAAGTATGATGG‐3’
	Reverse	5’‐GTTCCTGAGACGCTGTTC‐3’
TNF‐α (mouse)	Forward	5’‐CCGATGGGTTGTACCTTGTC‐3’
	Reverse	5’‐TGGAAGACTCCTCCCAGGTA‐3’
CXCL10 (mouse)	Forward	5’‐CCAAGTGCTGCCGTCATTTTC‐3’
	Reverse	5’‐GGCTCGCAGGGATGATTTCAA ‐3’
CCL5 (mouse)	Forward	5’‐ACCATGAAGATCTCTGCAGC‐3’
	Reverse	5’‐TGAACCCACTTCTTCTCTGG‐3’
GAPDH (human)	Forward	5’‐TGGTTGAGCACAGGGTACTT‐3’
	Reverse	5’‐CCAAGGAGTAAGACCCCTGG‐3’
GAPDH (mouse)	Forward	5’‐AGGTCGGTGTGAACGGATTTG‐3’
	Reverse	5’‐TGTAGACCATGTAGTTGAGGTCA‐3’

### RNA Sequencing (RNA‐seq) Assay and Data Analysis

For RNA‐seq analysis, THP‐1 cells were induced to differentiate into macrophages, followed by treatment with or without 4 µg mL^−1^ PVC NPs for 48 h. Three biological replicates were used in the study. Total RNA was extracted from the cells using TRIzol reagent. Library construction and sequencing were performed by LC‐Bio Technology Co. Ltd., Hangzhou, China.

### Plasmid Transfection and Viral Transduction

In accordance with the manufacturer's instructions, plasmid transfection was performed in HEK293T cells via the Jetprime transfection reagent kit (Polyplus, #101 000 046). The supernatant containing the lentivirus was collected at 48 and 72 h post‐transfection. This supernatant was used to infect THP‐1 cells seeded in a six‐well plate. After 48 hours of infection, puromycin (2 µg mL^−1^) (Beyotime, #ST551) was used for selection for 7 days. The gene knockout efficiency was then verified via western blotting.

### Immunofluorescence Staining

THP‐1 cells were cultured in 24‐well plates covered with glass slides. After treatment with 1 µm PMA, the cells were induced to adhere and differentiate into macrophages. The cells were then treated with PVC NPs for 48 h. After washing with PBS, the samples were fixed with 2% paraformaldehyde for 20 min and permeabilized with 0.5% PBST (0.15 mL Triton X‐100 + 50 mL PBS) (Macklin, #9002–93‐1) for 30 min. Prior to incubation with the primary antibody, non‐specific binding was blocked using an immunofluorescence blocking solution. The cells were then incubated overnight at 4 °C with the primary antibody (Anti‐DNA‐RNA Hybrid Antibody S9.6) (Antibody System, # RGK60001) in the dark. The following day, the glass slides were incubated with a secondary antibody (anti‐mouse IgG Alexa Fluor 488) (Invitrogen, # A‐10680) at room temperature in the dark for 1 h. Finally, the samples were mounted with antifade mounting medium containing DAPI (Beyotime, # P0131) and observed under a laser confocal microscope (Olympus, Japan) for imaging.

### Proximity Ligation Assay (PLA)

In this study, PLA was performed to detect the interaction between the R‐loop and RNASEH1 via the Duolink In Situ PLA Kit (Sigma‐Aldrich, # DUO92008) according to the manufacturer's instructions and previously reported methods.^[^ ^82^ ^]^ Briefly, cells overexpressing Flag‐RNASEH1, D210N mutant, or WKKD mutant, along with control cells, were plated on coverslips. Upon reaching the appropriate confluence, the samples were subjected to fixed, permeabilized, and blocked. The anti‐Flag primary antibody and S9.6 antibody were mixed in Duolink antibody diluent and incubated overnight at 4 °C. The next day, after washing with PBS, the PLA probes were incubated at 37 °C for 1 h, followed by ligation (30 min, 37 °C) and amplification (100 min, 37 °C) reactions. Finally, the samples were mounted with antifade mounting medium containing DAPI (Beyotime, #P0131), and images were acquired via a laser scanning confocal microscope (Olympus FV3000, Japan). PLA foci per cell were quantified via ImageJ software.

### Statistical Analysis

Statistical analysis was performed using GraphPad Prism 9.5 (GraphPad Software version 9.5.0) and SPSS 26.0 software. Comparisons between two groups were made via two‐tailed unpaired Student's *t*‐tests, and comparisons among multiple groups were made using one‐way or two‐way analysis of variance (ANOVA) followed by Tukey's multiple comparison test. The data were presented as the arithmetic mean ± SD. A value of *p* < 0.05 was considered to indicate statistically significant results, *p < 0.05, **p < 0.01, ***p < 0.001, ****p < 0.0001, NS, not significant. All experiments were performed with a minimum of three independent replicates.

## Conflict of Interest

The authors declare no conflict of interest.

## Author Contributions

Q.B. dealt with writing the original draft, methodology, validation, investigation, formal analysis, and data curation. Y.H. dealt with the methodology. M.D. dealt with the investigation. C.Z., D.Z., H.H., Y.Z., C.L., and Y.H. dealt with the validation. Y.H., H.L., and W.D. dealt with the software. Y.T. and J.J. dealt with the Resources. X.C. dealt with the supervision, resources, and project administration. Y.S. dealt with writing the review and editing, supervision, resources, funding acquisition, and conceptualization. Z.Y. dealt with writing the review and editing, supervision, resources, funding acquisition, and conceptualization.

## Supporting information



Supporting Information

## Data Availability

The data that support the findings of this study are available from the corresponding author upon reasonable request.
