# Supplementary material for: PVC Nanoplastics Exposure Exacerbates Asthma through R‐Loop Accumulation and Subsequent STING Activation in Macrophages
Source: Adv Sci (Weinh). 2025 Sep 8;12(44):e02223. doi: 10.1002/advs.202502223 (PMC12667527; doi:10.1002/advs.202502223)
Supplement: Supplementary file 1 — Supporting Information [file ADVS-12-e02223-s001.docx]

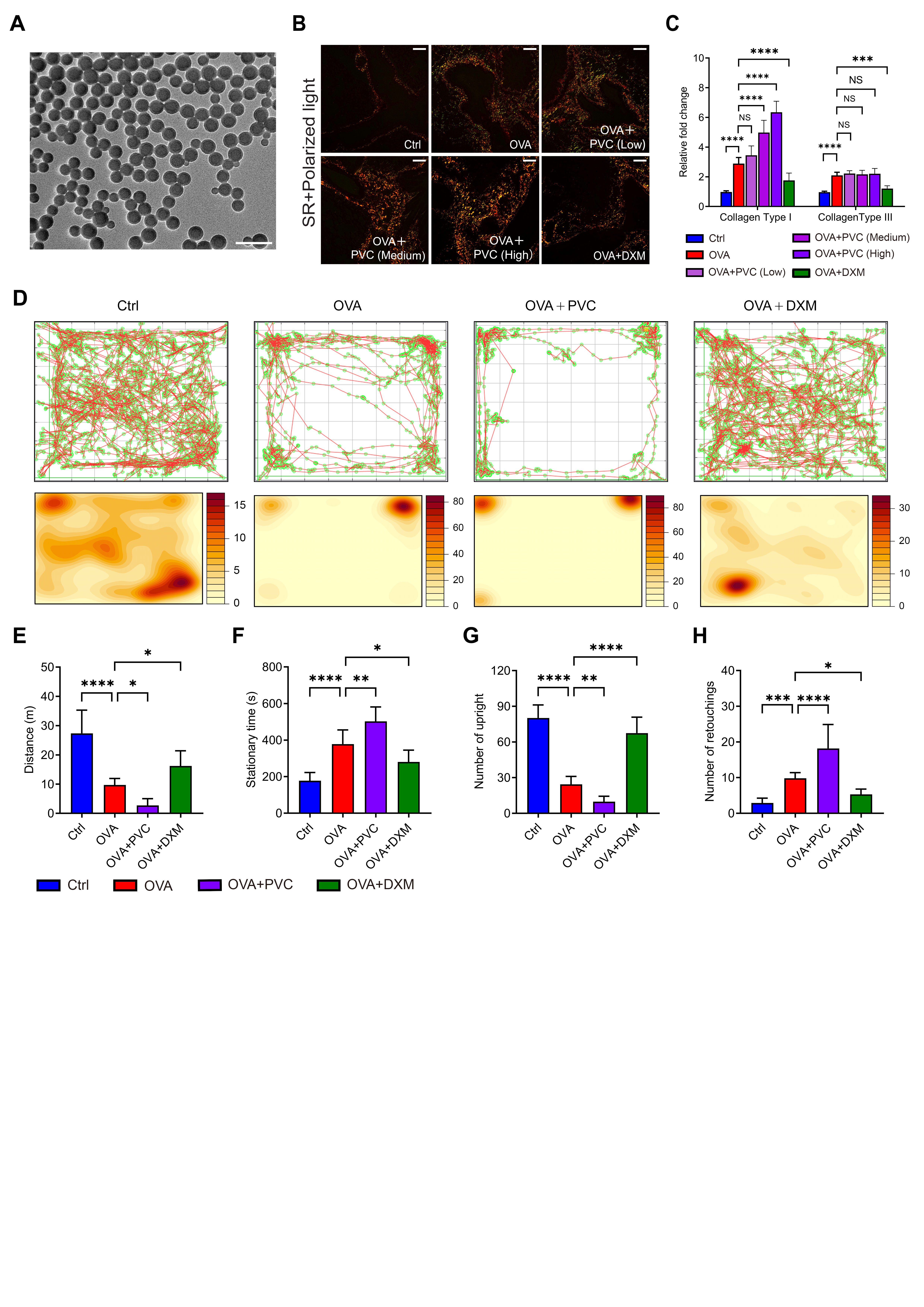


**Supplementary Figure S1.** Effects of PVC NPs on airway collagen deposition and behavioral parameters in OVA-induced allergic asthma mice. A) Representative TEM images depicting the morphology of the PVC NPs (scale bar = 100 nm). B-C) Representative images of Sirius red-stained mouse lung tissue sections from each group under polarized light microscopy (scale bar = 100 µm) and quantification of the distribution of different types of collagen fibers (n = 10). D) Representative trajectory plots and corresponding heatmaps of the mice from each group during the 10-minute recording period. The heatmaps indicate the time the animals spent in different parts of the body, with yellow indicating the shortest time and red indicating the longest time. E-H) Quantitative analysis of total travel distance (E), immobility time (F), rearing frequency (G), and grooming episodes in mice during the 10-minute test period (n = 10). Statistical analysis was performed using two-way (C) or one-way (E-H) ANOVA followed by Tukey's multiple comparison test. The data are presented as the mean ± SD; *p < 0.05, **p < 0.01, ***p < 0.001, ****p < 0.0001, NS, not significant.


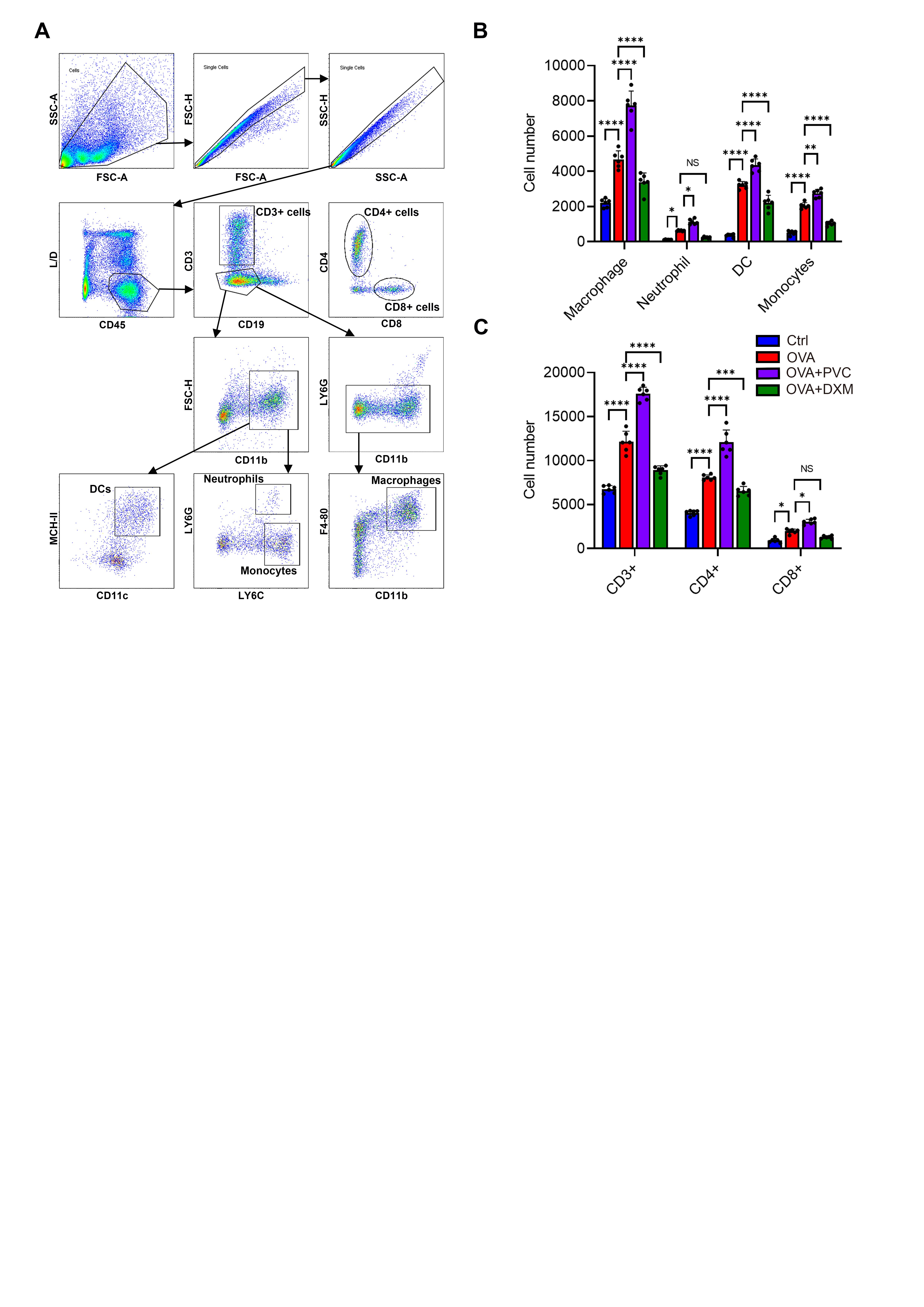


**Supplementary Figure S2.** Effects of PVC NPs on BALF immune cell profiles by flow cytometry. A) Gating strategy for flow cytometry. B-C) Flow cytometric analysis of macrophages, neutrophils, DCs, monocytes (B), and T cell subsets (C) in murine BALF (n = 6). Statistical analysis was performed using two-way ANOVA followed by Tukey's multiple comparison test. The data are presented as the mean ± SD; *p < 0.05, **p < 0.01, ****p < 0.0001, NS, not significant.


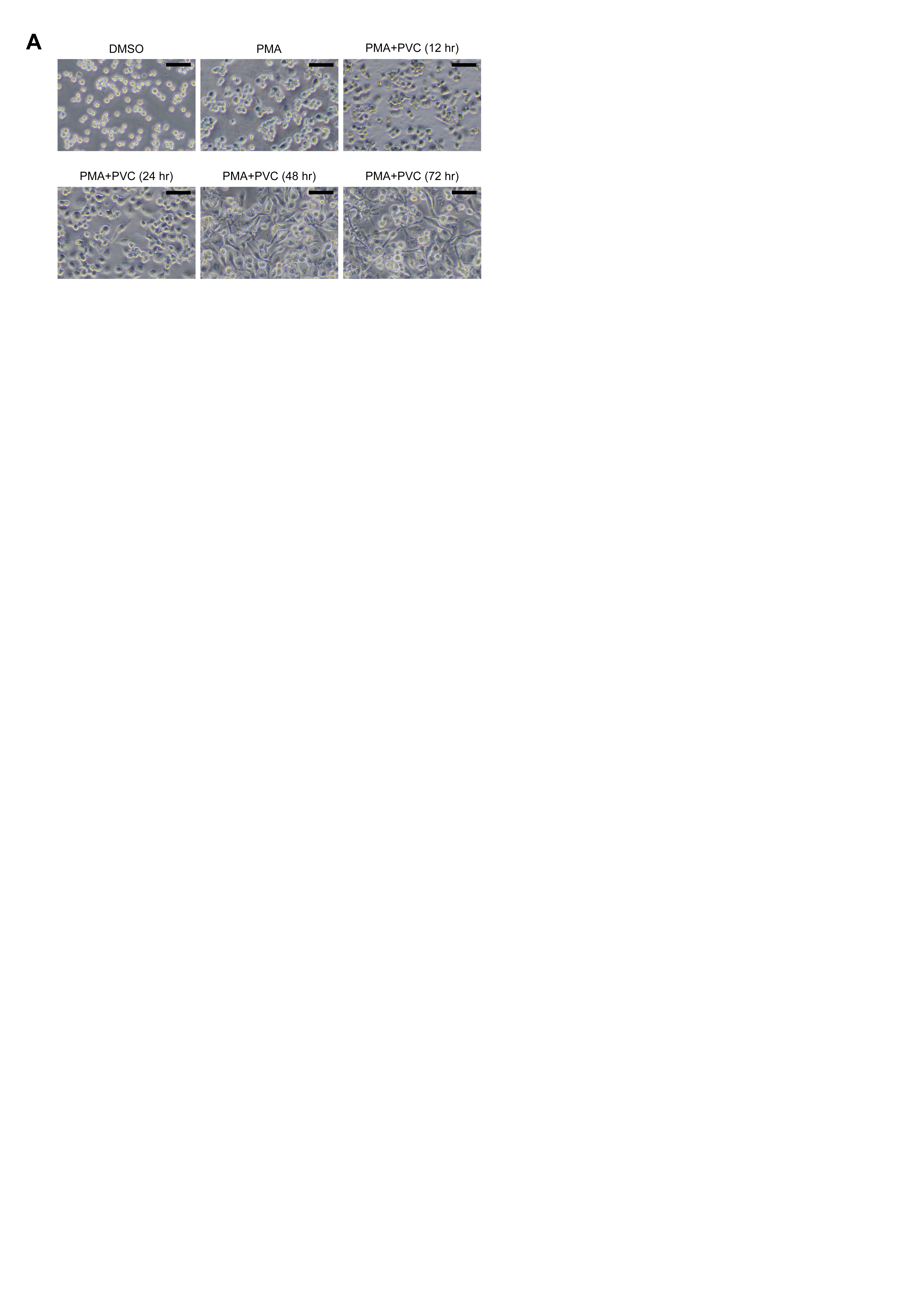


**Supplementary Figure S3.** Time-dependent morphological evolution of THP-1-derived macrophages following PVC NPs exposure. A) After THP-1 cells were induced to adhere and differentiate into macrophages with PMA, representative images of macrophage morphological changes were observed under a light microscope after treatment with PVC NPs for different time periods (scale bar = 20 µm).


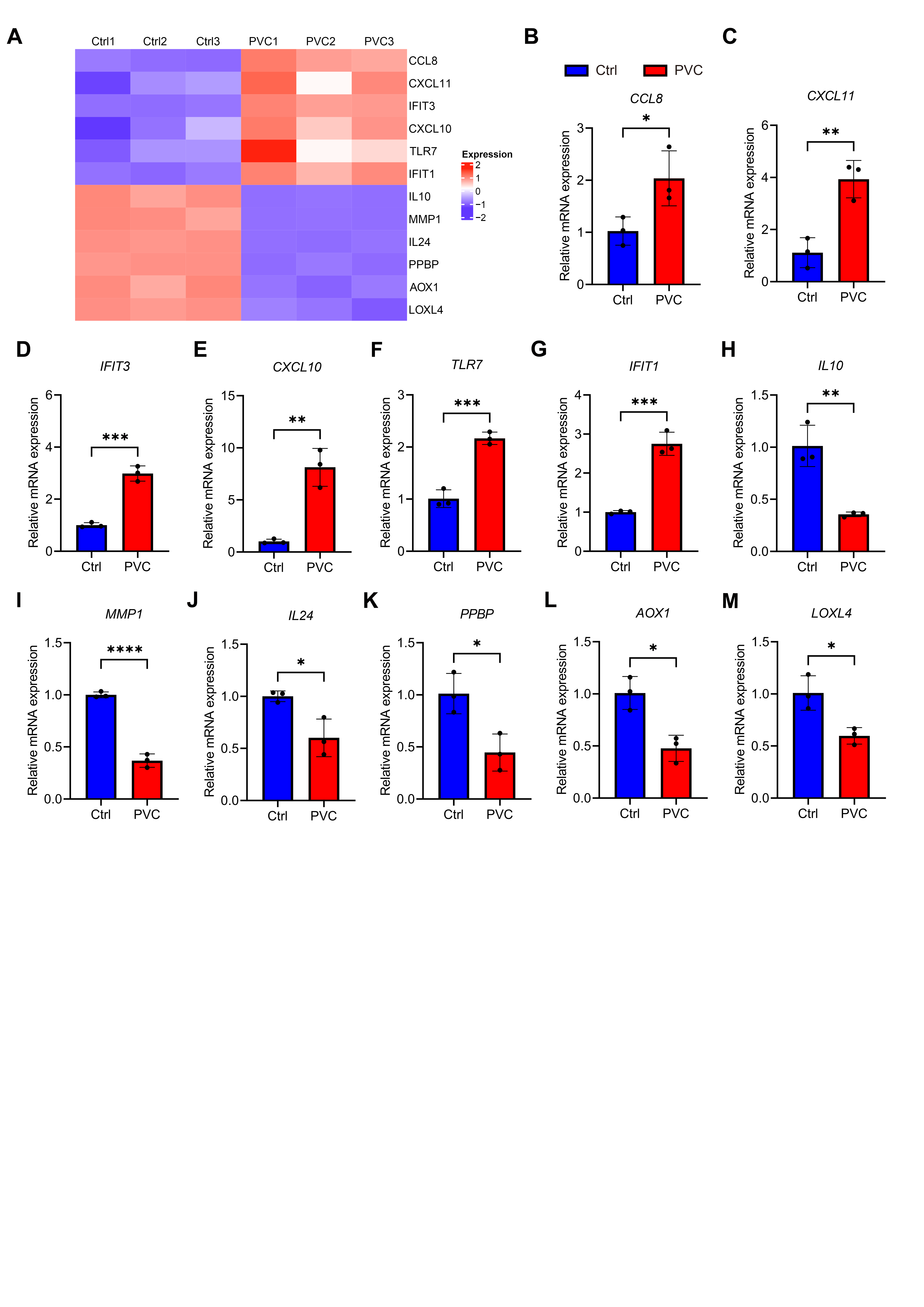


**Supplementary Figure S4.** Analysis of differentially expressed genes following PVC NPs exposure. A) Heatmap visualization of differentially expressed genes after PVC NPs exposure (red: upregulated; blue: downregulated). B-M) Validation of PVC NPs-induced differentially expressed genes via RT-qPCR (n = 3). Statistical analysis was performed using the unpaired two-tailed Student’s t-test. The data are presented as mean ± SD; *p < 0.05, **p < 0.01, ***p < 0.001, ****p < 0.0001.


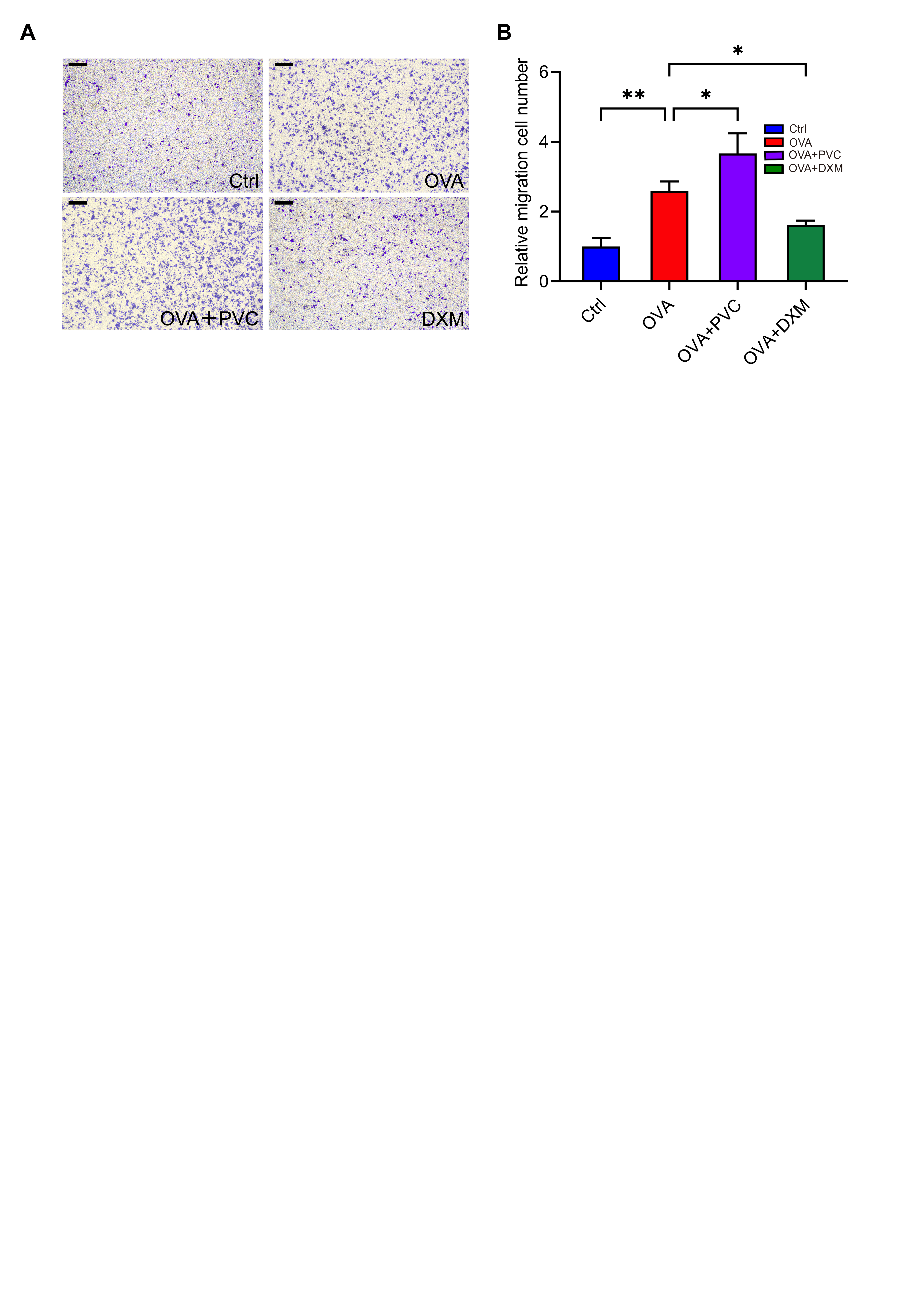


**Supplementary Figure S5.** Effect of BALF from PVC NPs-exposed asthmatic mice on macrophage migration. A) Representative images of macrophage migration assays treated with BALF from different asthmatic mice groups (scale bar = 300 µm). B) Quantification of migrated cells from three independent experiments. Statistical analysis was performed using one-way ANOVA followed by Tukey's multiple comparison test. The data are presented as mean ± SD; *p < 0.05, **p < 0.01.


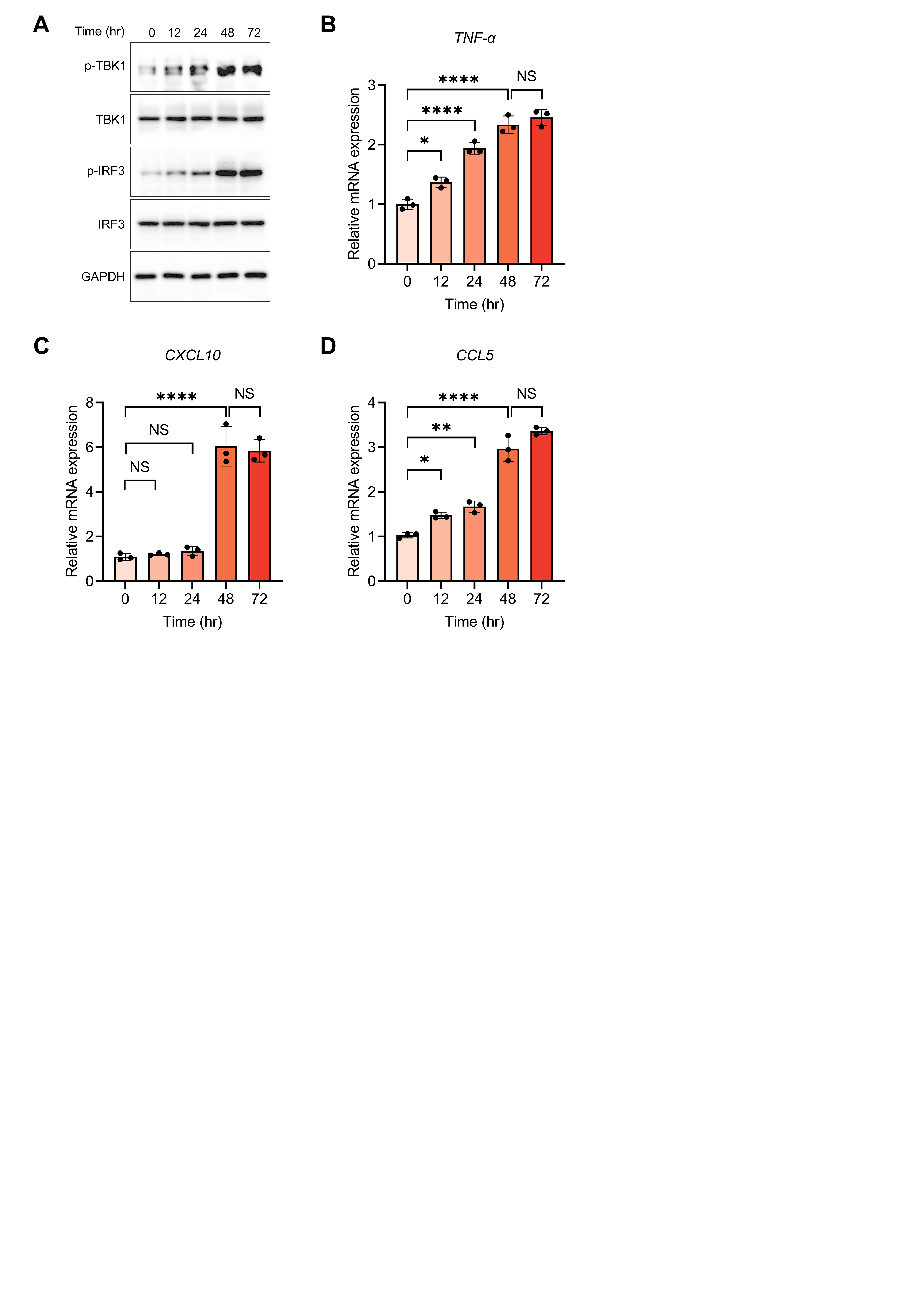


**Supplementary Figure S6.** PVC NPs time-dependently activate the cGAS-STING pathway. A) Time-dependent phosphorylation of TBK1 and IRF3 in THP-1-derived macrophages treated with 4 μg/mL PVC NPs by Western blot analysis. B-D) Relative mRNA expression levels of TNF-α (B), CXCL10 (C), and CCL5 (D) after PVC NPs treatment at different time points (n=3). Statistical analysis was performed using one-way ANOVA followed by Tukey's multiple comparison test. The data are presented as mean ± SD; *p < 0.05, **p < 0.01, ****p < 0.0001, NS, not significant.


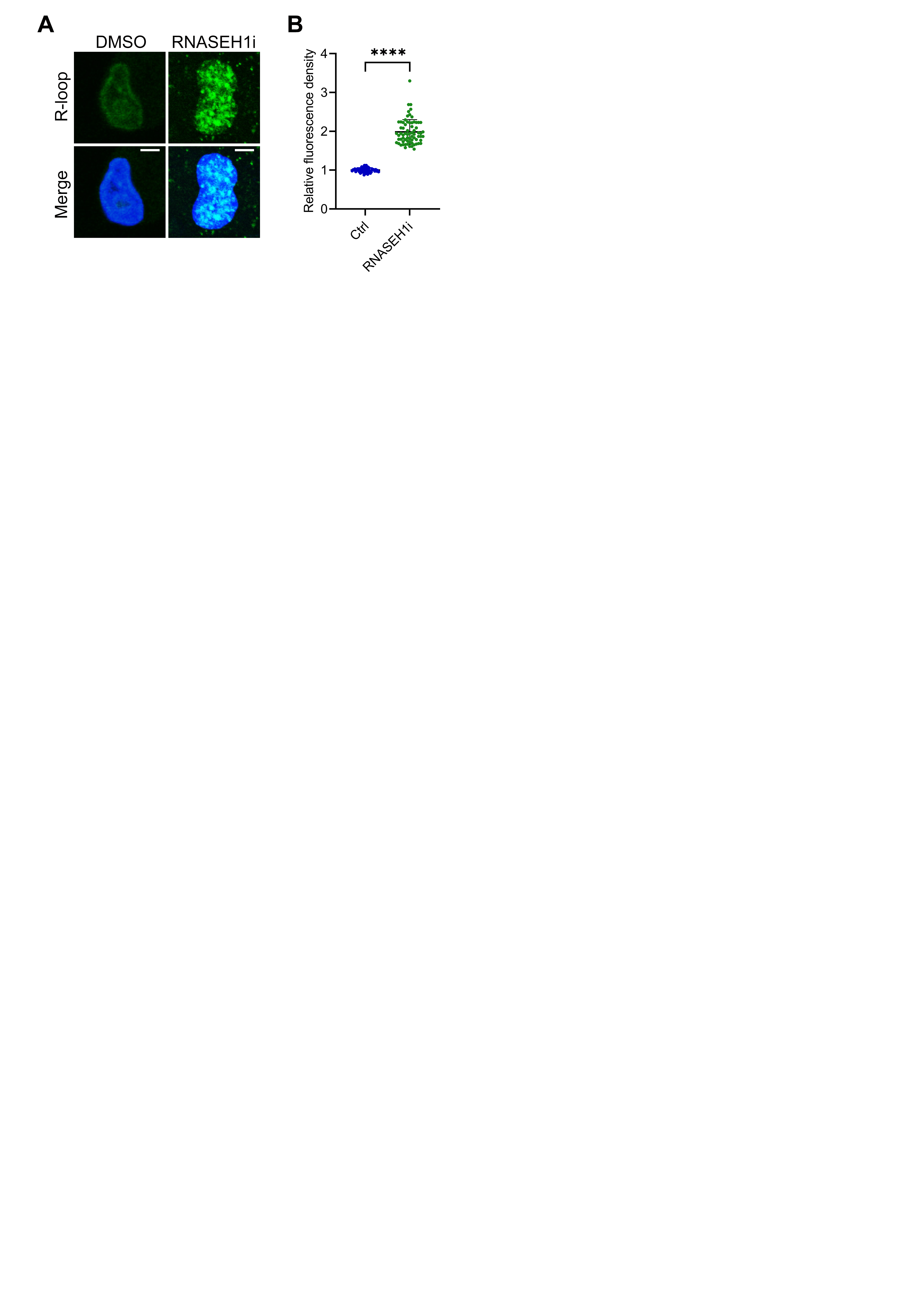


**Supplementary Figure S7.** RNASEH1-mediated suppression of R-loops. A) Representative composite fluorescence images showing the levels of R-loops after RNase H1 inhibitor treatment (scale bar = 3 μm). B) Quantitative analysis of the relative fluorescence intensity of R-loops in the nucleus of cells after RNase H1 inhibitor treatment (n>50 cells). Statistical analysis was performed using the unpaired two-tailed Student’s t-test. The data are presented as mean ± SD;****p < 0.0001.

**
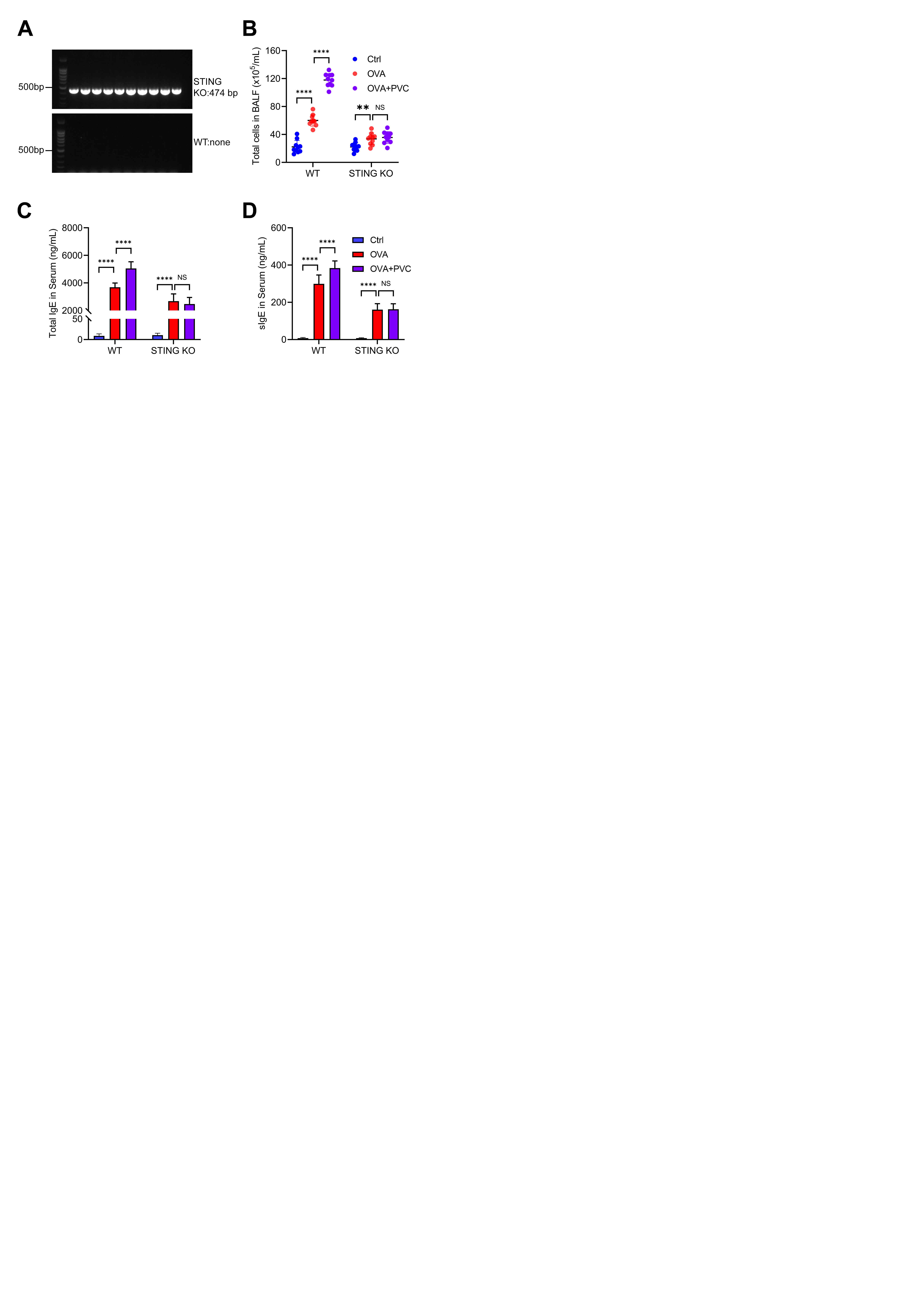
**

**Supplementary Figure S8.** STING deletion attenuates PVC NPs-induced inflammation in asthmatic models. A) Genotyping results for the STING KO allele. The STING KO is 474 bp (top), and the WT allele is none (below). B) Quantitative analysis of the total number of inflammatory cells in the BALF from each group of mice (n = 10). C-D) Quantitative analysis of total IgE and sIgE concentrations in the serum of each group of mice via ELISA (n = 10). Statistical analysis was performed using two-way ANOVA followed by Tukey's multiple comparison test. The data are presented as mean ± SD; **p < 0.01, ****p < 0.0001, NS, not significant.
